# Single cell RNA-sequencing identified *Dec2* as a suppressive factor for spermatogonial differentiation by inhibiting *Sohlh1* expression

**DOI:** 10.1038/s41598-019-42578-z

**Published:** 2019-04-15

**Authors:** Yoshinori Makino, Niels H. Jensen, Naoko Yokota, Moritz J. Rossner, Haruhiko Akiyama, Katsuhiko Shirahige, Yuki Okada

**Affiliations:** 1 Laboratory of Pathology and Development, Institute for Quantitative Biosciences, The University of Tokyo, 1-1-1 Yayoi, Bunkyo-ku, Tokyo, 113-0032 Japan; 2 Molecular and Behavioral Neurobiology, Department of Psychiatry and Psychotherapy, University Hospital, LMU Munich, Munich, Germany; 3 Laboratory of Genome Structure and Function, the Institute for Quantitative Biosciences, University of Tokyo, 1-1-1 Yayoi, Bunkyo, Tokyo, 113-0032 Japan; 4 Department of Orthopaedic Surgery, Gifu University Graduate School of Medicine, Yanagido 1-1, Gifu, 501-1194 Japan

## Abstract

Gonocyte-to-spermatogonia transition is a critical fate determination process to initiate sperm production throughout the lifecycle. However, the molecular dynamics of this process has not been fully elucidated mainly due to the asynchronized differentiation stages of neonatal germ cells. In this study, we employed single cell RNA sequencing analyses of P1.5–5.5 germ cells to clarify the temporal dynamics of gene expression during gonocyte-to-spermatogonia transition. The analyses identified transcriptional modules, one of which regulates spermatogonial gene network in neonatal germ cells. Among them, we identified *Dec2*, a bHLH-type transcription factor, as a transcriptional repressor for a spermatogonial differentiation factor *Sohlh1*. Deficiency of *Dec2* in mice induces significant reduction of undifferentiated spermatogonia, and transplantation assay using *Dec2*-depleted cells also demonstrated the impaired efficiency of engraftment, suggesting its role in maintaining spermatogonial stem cells (SSCs). Collectively, this study revealed the intrinsic role of a new SSC factor *Dec2*, which protects germ cells from inadequate differentiation during neonatal testis development.

## Introduction

Spermatogenesis is a process to produce spermatozoa almost throughout the lifecycle, and sustained by spermatogonial stem cells (SSCs) that can both self-renew and differentiate^1–3^. In mouse testis, embryonic male germ cells called gonocytes/prospermatogonia, which are the sole source of a functional reservoir of SSCs, resume cell proliferation at around postnatal day (P) 1.5–2 and migrate from the center of the seminiferous tubules to the basement membrane side at around P3. By P4–5, germ cells start to be subjected to two alternative cell fates: SSCs or type A spermatogonia, the latter of which commit further differentiation to initiate the first wave of spermatogenesis^3,4^. It’s known that germ cells in this period, also known as “gonocyte-to-spermatogonia transition”, exhibit remarkable heterogeneity based on their marker gene expressions, while it is difficult to distinguish whether this heterogeneity is caused by different populations of germ cells or simply due to their asynchronized developmental stages^3,5–7^.

To solve such a problem, single cell RNA sequencing (scRNA-seq), a systematic approach independent of specific markers, has become a powerful tool for characterizing heterogenous cell populations. To date, several scRNA-seq studies have attempted to dissolve the temporal transition of cellular state as well as the heterogeneity of undifferentiated spermatogonia in both mouse and human^8–15^. In particular, more recent studies successfully captured the temporal transcriptional dynamics during spermatogonial development by combining single cell transcriptome and pseudotime analyses, further confirming the effectiveness of single cell analyses for asynchronized cell populations^8,9,12,14^. In these studies, it was clearly demonstrated that the temporal transition from SSC/undifferentiated to differentiating state of spermatogonia are characterized by the transition from higher expressions of *Id4*, *Gfra1, Etv5*, and *Lhx1* to *Sohlh1/2*, *Kit*, and *Star8* as numbers of former studies have been demonstrated by immunostaining, RT-PCR, etc.^16–23^. However, although this transition is physiologically induced by retinoic acid (RA) stimulation, the underlying molecular pathway has not been fully elucidated.

*Sohlh1* belongs to basic helix-loop-helix (bHLH) transcription factor (TF) family. In general, bHLH TFs play important roles in cellular differentiation during the various developmental stages of organogenesis^24–29^. Some of the bHLH factors express and function in tissue-specific manner, and *Sohlh1* was first identified as a testis-specific bHLH factor that is strongly expressed from type A_al_ to type B spermatogonia^20,21^. In mice, its deficiency causes impaired differentiation of spermatogonia, suggesting its critical role for spermatogonial differentiation^20,21^. *Sohlh1* forms a heterodimer with its paralogue *Sohlh2*^21^, and the dimer contributes to auto-activate *Sohlh1* by binding to the E-box motifs on its own promoter^30^. As described above, expression of SOHLH1 is induced in the response of RA as early as P3–4 in neonatal mouse testis. It’s recently demonstrated that the induction of SOHLH1 in neonatal testis by RA is through PI3K/AKT/mTOR-dependent translational regulation rather than transcriptional activation^31^. Therefore, the transcriptional control of *Sohlh1* in neonatal testis remained to be investigated.

In the present study, we performed scRNA-seq using neonatal male germ cells to search the factors and transcriptional networks involved in gonocyte-to-spermatogonia transition. Notably, we identified several TFs as possible SSC factors. Among them, we focused on a bHLH transcription repressor *Dec2/Sharp1/Behlh41*, which is known to be a clock gene and a repressor of the differentiation of mesenchymal stem cells^32–40^. We experimentally demonstrated that *Dec2* plays an inhibitory role in spermatogonial differentiation in neonatal germ cells by suppressing *Sohlh1* expression.

## Results

### scRNA-seq of neonatal germ cells demonstrates pseudotime-dependent transcriptional dynamics

To more precisely understand gonocyte-to-spermatogonia transition from the perspective of cellular heterogeneity and temporally regulated gene expression patterns, male germ cells were collected from P1.5, P3.5, and P5.5 testes and subjected to scRNA-seq. For isolating germ cells, we used a transgenic mouse line exhibiting germ cell-specific expression of histone H4-Venus fusion protein^41^. After cell sorting, Venus(+) cells were subjected to scRNA-seq analyses, and sequence data could be recovered from 177 cells. After primary data processing, we first calculated the correlation between the number of mapped reads and expressed genes to estimate how many reads from a single cell could represent the global trend under this experimental design. The result indicated that two million reads were required to capture the global trend (Fig. S1a), while in some cells, the number of reads did not reach two million. Therefore, one million reads were randomly extracted from each cell for the subsequent quality check to eliminate samples with improper Transcript Per Million (TPM) values (Fig. S1b). As a result, two cells were eliminated and the remaining 175 cells (80 cells from P1.5, 48 cells from P3.5, and 47 cells from P5.5, respectively) were subjected to further bioinformatical analyses.

**Figure 1:**
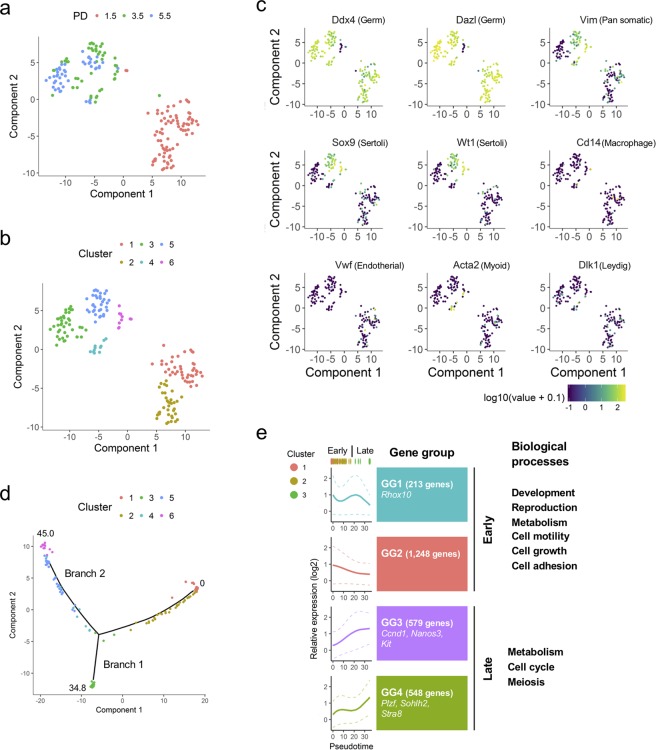
scRNA-seq analysis depicts the developmental processes of neonatal germ cells. (**a,b**) t-SNE plots of single cell transcriptome data from P1.5, P3.5, and P5.5 cell samples. Clustering analysis (**b**) was performed on t-SNE plot by all the expressed genes. (**c**) Expression patterns and levels of selected marker genes projected on the t-SNE plots. Purple and yellow indicate lower and higher expressions, respectively. (**d**) Construction of pseudotime-trajectory plot calculated with Monocle software. The values of pseudotimes were indicated at the beginning of the trajectory (0) and the ends of branches (34.8 in Branch 1 and 45.0 in Branch 2). Clusters 1–6 shown in (**b**) were projected on the trajectory in colors as indicated. (**e**) Expression dynamics of the gene groups. Pseudotime DEGs were divided into four gene groups (GG1 to GG4) depending on their expression dynamics. Note that only Clusters 1–3 were applied to this analysis. The graphs of expression patterns of GG1 to GG4 were shown on the left side. Pseudotime attributions of Clusters 1–3 cells were depicted in colors as indicated at the upper side of the graphs. Solid and dashed lines indicate pseudotime-fitted curves of the average and standard deviations, respectively. The representative genes and enriched gene ontology terms of the gene groups were described on the right side.

From the 175 cells, 13,514 genes were successfully captured (Table S1, Fig. S2a). The plot of t-Distributed Stochastic Neighbor Embedding (t-SNE) depicted that P1.5 cells formed a distinct population from P3.5 and P5.5 cells, while P3.5 and P5.5 cells were inseparable from each other (Fig. 1a). Clustering analysis on the t-SNE plot divided the cells into six distinct clusters designated as Clusters 1 to 6 (Figs 1b, S2b). Clusters 1 to 5 expressed pan-germ cell markers *Ddx4* and *Dazl* as well as 22 marker genes, the expressions of which were demonstrated in gonocytes and/or spermatogonia, in various extents (Figs 1c, S2c)^5,6,42,43^. In contrast, Cluster 6 expressed somatic cell markers such as *Vim, Sox9*, *Gata4*, and *Wt1* without expressing *Ddx4* and *Dazl*^8,44–48^, indicating that Clusters 1 to 5 were germ cells and Cluster 6 was contaminated somatic cells (Figs 1c, S2c). In addition to Cluster 6, Cluster 4 and 5 also expressed somatic cell markers (*Vim* in both Cluster 4 and 5; *Sox9, Gata4*, and *Wt1* in Cluster 5; *Acta2* in Cluster 4) as well as the germ cell markers (Figs 1c, S2c). These miscellaneous clusters caused the trajectory path analysis combined with pseudotime to depict a bifurcated path, which started from Cluster 1 and reached to the bifurcation point *via* Cluster 2, and separated into two endpoints as branch 1 (Cluster 3) and branch 2 (Cluster 4 to 6), respectively (Figs 1d, S4a, S4b). Since a previous study has also reported miscellaneous expressions of Sertoli cell markers *Sox9* and *Wt1* in postnatal male germ cells by single cell RT-qPCR^15^, we tried to characterize the ambiguous cell population in Cluster 5. However, immunostaining of P4.5 testes clearly showed the restricted expression of SOX9 protein only in Sertoli cells (Fig. S3a). To exclude the possibility of unstable expression of SOX9 protein in germ cells, we further employed Sox9-EGFP mice, in which an IRES-EGFP-poly A cassette was inserted into the 3′-untranslated region of the *Sox9*. The Sox9-EGFP mice were crossed with Vas-RFP transgenic mice, in which RFP was specifically expressed in germ cells by *Vasa/Mvh* promoter. P5.5 testes of the male progenies heterozygously carrying both Sox9-EGFP and Vas-RFP were subjected to flow cytometry analysis. Consistent to the immunostaining, it also failed to detect EGFP-expressing germ cells (Fig. S3b). Similar to *Sox9*, another Sertoli cell marker *Gata4* also exhibited higher mRNA expression in Cluster 5^47,48^, while the protein expression was undetectable in PLZF(+) undifferentiated spermatogonia in P5.5 testes (Fig. S3c). Collectively, we were unable to characterize the Sertoli-like germ cell populations in Cluster 5. Cluster 4 was also miscellaneous due to the apparent expression of a myoid cell marker *Acta2*. Thus, we eliminated these two clusters as well as Cluster 6 (somatic cells) and applied Clusters 1 to 3 (119 cells) into subsequent analyses.

Next, we analyzed pseudotime-fitted gene expression dynamics in Clusters 1–3 and identified 2,588 genes as “differently expressed genes along the pseudotime (= pseudotime DEGs, *q* < 0.05)” (Table S2). Further hierarchical clustering analysis divided the pseudotime DEGs into four groups [Gene group (GG) 1 to 4]: GG1 (213 genes), GG2 (1,248 genes), GG3 (579 genes), and GG4 (548 genes), respectively (Figs 1e, S4c, Table S2). When we divided the dynamics of pseudotime into “early” (<18, mainly composed by Clusters 1 and 2) and “late” (>18, mainly composed by Cluster 3), GG1 and GG2 were characterized by the enrichment of early expressed genes, while genes in GG3 and GG4 were preferentially expressed in the late period (Figs 1e, S4c). According to the gene ontology (GO) analysis, GG1 and GG2 genes were significantly related to development, reproduction, metabolism, cell motility, cell growth, and cell proliferation, while GG3 and GG4 late expressed genes were associated to metabolism, cell cycle, and meiosis (Fig. 1e and Table S3). These findings were consistent to the idea that quiescent gonocytes (a.k.a. T1 prospermatogonia) resume cell division at around P2, move to the basal layer of seminiferous tubules, and become spermatogonia, which undergo rapid proliferation^3,5–7^.

We next examined the expressions of known 24 gonocyte/spermatogonial genes shown in Fig. S2c to verify the relevance of our cell clustering and pseudotime analyses in Clusters 1–3 (Fig. S5 and Table S4). Expressions of undifferentiated spermatogonial markers *Gfra1*, *Ret*, and *Id4*^16,17^ and a cell cycle regulator *Ccnd2*^49^ reached the highest levels in the early pseudotime and maintained their expressions until the late pseudotime period (Figs S2c, S5 and Table S4). Another set of marker genes for undifferentiated spermatogonia and/or gonocyte-to-spermatogonia transition *Bmi1* and *Rhox10*^13,50^ reached the highest expression levels at the onset of peudotime (= pseudotime 0) (Figs. S2c, S5 and Table S4). In contrast, expressions of differentiating/differentiated spermatogonial markers such as *Sohlh1, Sohlh2*, *Kit*, and *Stra8*^20–23^ and cell cycle regulators *Ccnd1 and Mycn*^49,51^ became prominent in the later pseudotime (Figs S2c, S5 and Table S4), suggesting that our clustering and pseudotime analyses reflected their endogenous expression patterns accordingly. There were also a few exceptions such as *Nanos2, Neurog3, Pax7*, and *Thy1*, the expression dynamics of which were failed to determine because of being detected in a very small portion of cells most likely due to the insufficient sequencing depth (Figs S2c, S5). Collectively, we concluded that Clusters 1, 2, and 3 represented quiescent gonocytes, migratory/mitotic gonocytes (a.k.a. T2 prospermatoaonia), and spermatogonia, respectively. Interestingly, we also noticed transient and subtle expressions of differentiating spermatogonial markers *Sohlh1, Sohlh2, and Kit*^20–22^ in the beginning of pseudotime (i.e. in Cluster 1/quiescent gonocytes), although their major expressions occurred in the later pseudotime (Figs S2c, S5), suggesting that transcription of these differentiation markers is activated at certain levels in quiescent gonocytes and subsequently suppressed until the substantial expressions are required in differentiating spermatogonia.

### Transcription factor network analysis identifies a transcription factor network module that possibly orchestrates neonatal spermatogonia gene expressions

**Figure 2:**
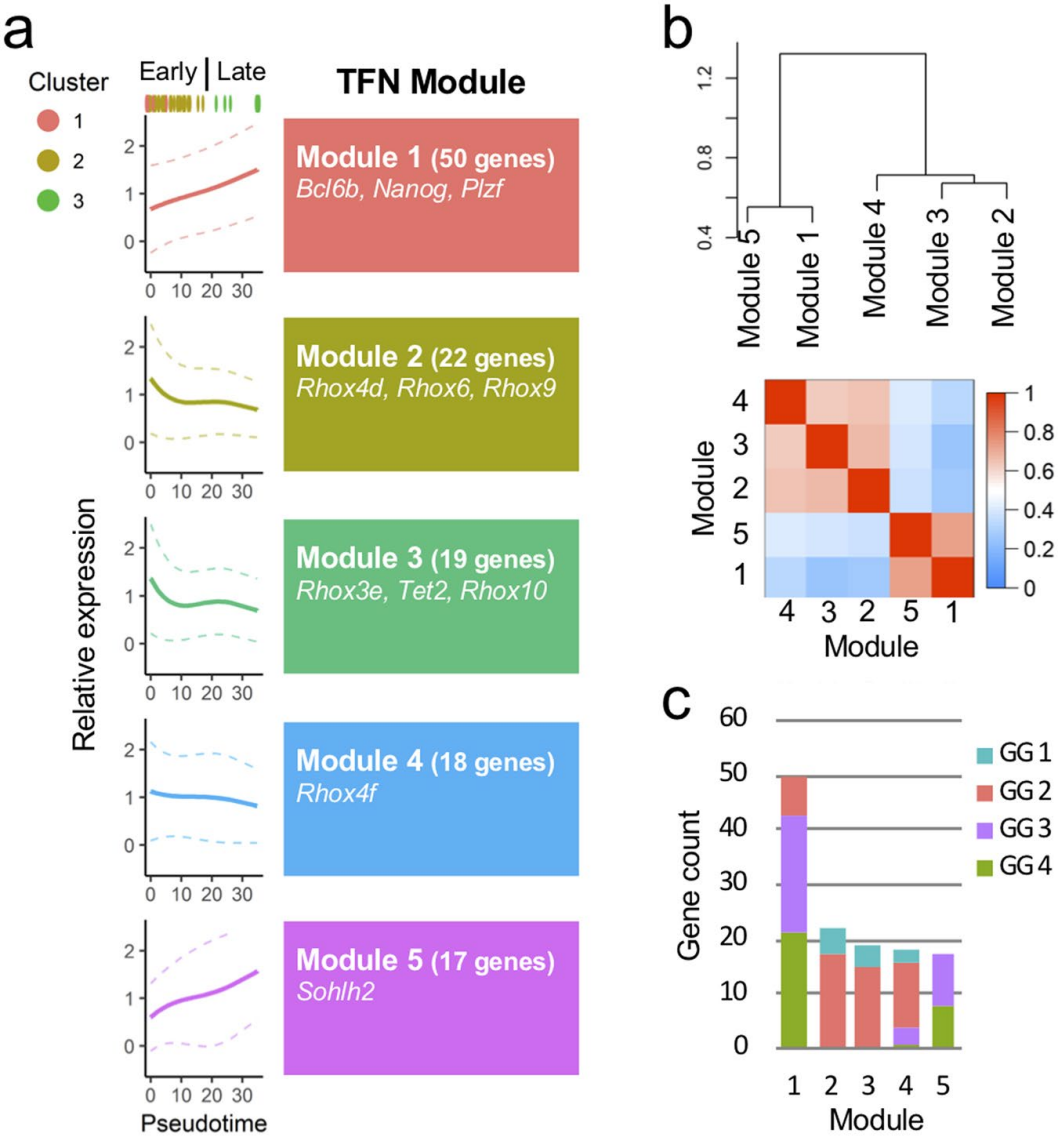
WGCNA identifies transcription factor network (TFN) modules potentially involved in neonatal germ cell development. (**a**) Five distinct TFN modules were identified by expression correlation analysis. Solid and dashed lines indicate pseudotime-fitted curves of the average and standard deviations, respectively. (**b**) Hierarchical dendrogram (upper panel) and topological overlap matrix (lower panel) of the TFN modules. Red and blue in the heatmap denote higher and lower correlations, respectively. (**c**) Gene counts in each TFN Module. Their attributes in the gene groups (GG1 to GG4) are also indicated.

Since temporally regulated gene expression patterns implied that precise transcription factor networks (TFNs) were involved in neonatal germ cell development and differentiation, we further performed weighted gene co-expression network analysis (WGCNA). For this purpose, 201 pseudotime DEG TFs were subjected to the WGCNA. The result identified five cohesive TFN modules (Modules 1–5, Figs 2a, b, S6a, Table S5). In Modules 1 and 5, the TFs exhibited preferential expressions in the late pseudotime, while Modules 2, 3, and 4 TFs were gradually decreased their expression levels as pseudotime proceeded (Fig. 2a). The GO analysis found that in both Modules 1/5 and 2/3/4, the GO terms of “metabolic processes” and “developmental process” were significantly enriched (Table S6). Additionally, the term of “cell proliferation” was enriched in Modules 2/3/4 (Table S6). Consistent with their expression dynamics, Modules 1 and 5 were characterized by predominant occupancy of GG3 and GG4 late genes, whereas Modules 2, 3, and 4 mainly possessed GG1 and GG2 early genes (Figs. 2b, c). Collectively, we concluded that Modules 1/5 and 2/3/4 corresponded to spermatogonial and gonocyte TFN modules, respectively. Interestingly, although the majority was GG3 and GG4, seven GG2 early genes were included in a “spermatogonial” TFN Module 1 (Figs. S6b, c). Since many SSC factors belong to GG2 (Fig. S5), we assumed that these seven TFs participated in transcriptional regulation of undifferentiated property. Among them, we further focused on *Dec2* because previous studies uncovered an inhibitory effect of *Dec2* for differentiation-related factors in other types of stem cells^38–40^.

### *Dec2* is preferentially expressed in early undifferentiated spermatogonia

Since the role of DEC2 in germ cells has not been reported, we first generated an antibody against DEC2 to verify its expression in perinatal and neonatal spermatogenesis. Western blotting analysis demonstrated that the antibody successfully recognized not only overexpressed Flag-tagged DEC2, but also the endogenous protein in various mouse tissues (Fig. S7a). Endogenous DEC2 was recognized at two different sizes in most tissues, suggesting the possibility of either splicing variants or modifications depending on tissue types (red and blue arrowheads, Fig. S7a). Importantly, the antibody absorption test by recombinant DEC2 resulted in a remarkable reduction of DEC2 signals (Fig. S7b), indicating the specificity of our antibody. Next, we performed immunohistochemistry of P5.5 testes to confirm the expression of DEC2 in germ cells. The results indicated that most of the DEC2(+) cells expressed a pan-germ cell marker GENA/TRA-98 (yellow arrows; Fig. S8)^52^, and these signals were greatly reduced when the antibody was pre-absorbed by recombinant DEC2 (Fig. S8). Although we occasionally observed DEC2(+) non-germ cells (white arrowheads, Fig. S8, middle panels), they were all mitotic, and the signals were retained even when the absorbed antibody was used (white arrowheads, Fig. S8, lower panels), implying its non-specific reaction against mitotic cells. Although we cannot deny the possibility that DEC2 does express in mitotic cells, we, therefore, excluded these cells from the following quantification of immunohistochemical analyses.

**Figure 3:**
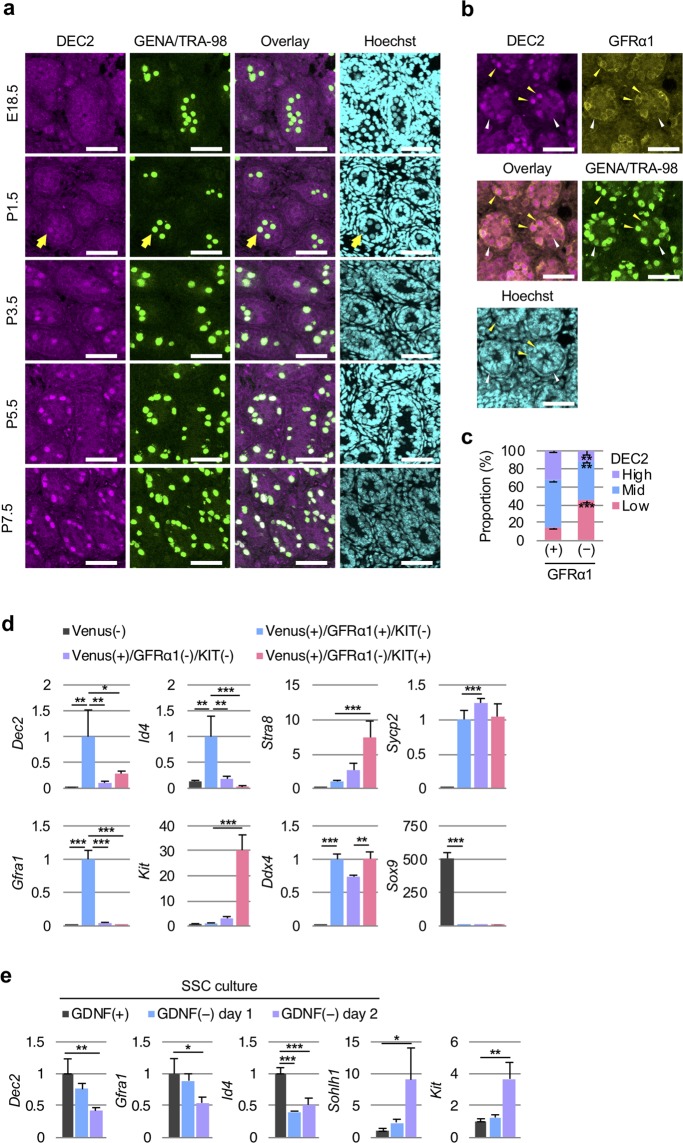
*Dec2* is highly expressed in GFRα1(+) undifferentiated germ cells in neonatal testes. (**a**) Immunohisto-chemical analyses of DEC2 expression in E18.5–P7.5 testes. Yellow arrows in P1.5 panels indicate a germ cell expressing DEC2. Scale, 50 μm. (**b**) Co-existence of DEC2 and GFRα1 in P7.5 testes. Yellow arrowheads indicate DEC2^high^/GFRα1(+) germ cells. White arrowheads indicate DEC2^low^/GFRα1(−) germ cells. Scale, 50 μm. (**c**) Quantitative analysis of the images shown in (**b**). Student’s t-test was performed for statistical analysis (three biological replicates). (**d**) Expression profiles of *Dec2* and selected marker genes in different cell populations in P7.5 testes. H4-Venus was used to isolate germ cells. After cell sorting, gene expression levels in each cell population were quantified by RT-qPCR. The expression levels of H4-Venus(−) (somatic) samples were designated as the value of 1. Dunnett’s test against H4-Venus(+)/GFRα1(+)/KIT(−) samples (blue bars) was performed for statistical analysis (three biological replicates). (**e**) Expression profiles of *Dec2* and marker genes in cultured SSCs in the absence of GDNF. Their expression levels were quantified by RT-qPCR. Expression levels of GDNF(+) samples (black bars) were designated as the value of 1. Dunnett’s test against GDNF(+) samples was performed for statistical analysis (three biological replicates). GENA/TRA-98, a pan-germ cell marker; Hoechst, DNA staining. Error bars indicated standard deviations. *p value < 0.05; **p value < 0.01; ***p value < 0.001.

Further immunostaining of E18.5–P7.5 testes demonstrated germ cell-specific expression of DEC2, which started in P1.5 (yellow arrow, Fig. 3a) and became prominent at P3.5 (Fig. 3a). Immunostaining of P7.5 testes also indicated that DEC2 protein levels were significantly higher in GFRα1(+) undifferentiated spermatogonia, compared with those in GFRα1(−) spermatogonia (Figs. 3b, c), suggesting the role of DEC2 in GFRα1(+) undifferentiated spermatogonia including SSCs. To confirm the enrichment of *Dec2* transcripts in the GFRα1(+) population, we next divided P7.5 testicular cells into H4-Venus(−) (somatic cells), H4-Venus(+)/GFRα1(+)/KIT(−) (early undifferentiated spermatogonia), H4-Venus(+)/GFRα1(−)/KIT(−) (late undifferentiated spermatogonia), and H4-Venus(+)/GFRα1(−)/KIT(+) (differentiating spermatogonia) by cell sorting (Fig. S9). The RT-qPCR analysis confirmed the technical relevance of sorting by known marker gene expressions and an enriched expression of *Dec2* in early undifferentiated spermatogonia (Fig. 3d). We also tested the change of *Dec2* expression in GDNF-depleted primary cultured SSCs to see whether *Dec2* expression was regulated by the GDNF/GFRα1 signaling pathway. The result indicated time-dependent substantial reductions of *Dec2* by GDNF withdrawal similar to other undifferentiated markers *Gfra1* and *Id4*, while the early differentiation markers *Sohlh1* and *Kit* were inversely upregulated (Fig. 3e).

### *Dec2* deficiency causes reduced SSCs

**Figure 4:**
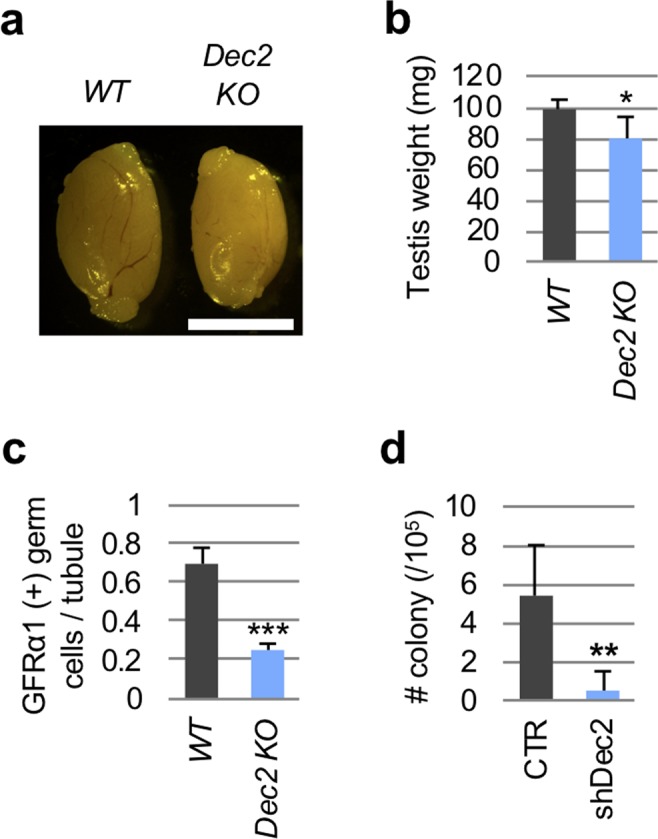
*Dec2* depletion causes the reduced GFRα1(+) undifferentiated spermatogonia. (**a**) Gross image of adult testes from wild type and *Dec2* knockout mice. WT, wild type; KO, *Dec2* knockout. Scale, 5 mm. (**b**) Testicular weights of WT (n = 5) and KO (n = 3) were measured. (**c**) Number of GFRα1(+) germ cells were counted in the testicular sections of WT (n = 5) and KO (n = 3). (**d**) SSC transplantation analysis. Donor-derived H4-Venus(+) colonies were counted eight weeks after transplantation in control (CTR, n = 5) and shDec2 (n = 4) samples. Student’s t-test was performed for statistical analysis. Error bars indicated standard deviations. *p value < 0.05; **p value < 0.01; ***p value < 0.001.

To further confirm the importance of DEC2 in spermatogenesis *in vivo*, we investigated the testicular phenotype of *Dec2* knockout (KO) male mice^53^. Although *Dec2* KO males were fertile, and histological features of spermatogenesis overall were maintained (Fig. S10), the testicular weights were slightly but significantly reduced (Figs. 4a,b), and the numbers of GFRα1(+) undifferentiated spermatogonia dropped to 36% of the control samples in the *Dec2* KO testes (Fig. 4c). By transplantation analysis, we further attempted to confirm whether reduced numbers of GFRα1(+) cells in *Dec2* KO mice were due to the intrinsic effects of male germ cells. For this purpose, three different shRNAs against *Dec2* (shDec2 #1–3) were designed and introduced into cultured SSCs using lentiviruses, which achieved >90% infection efficiencies (Figs. S11a, b). This approach resulted in 65–95% knockdown efficiencies, while cell viabilities were well maintained (Figs. S11c, d). We used shDec2 #1 for further experiments due to its highest knockdown efficiency (~95%). The transplantation assay demonstrated significantly reduced donor cell-derived colonies by *Dec2* knockdown (Fig. 4d). This was consistent with observations of *Dec2* KO testis and further supported the idea that DEC2 functions to maintain SSCs.

### DEC2 suppresses the transcription of *Sohlh1* by competing with SOHLH1 when binding to E-box

**Figure 5:**
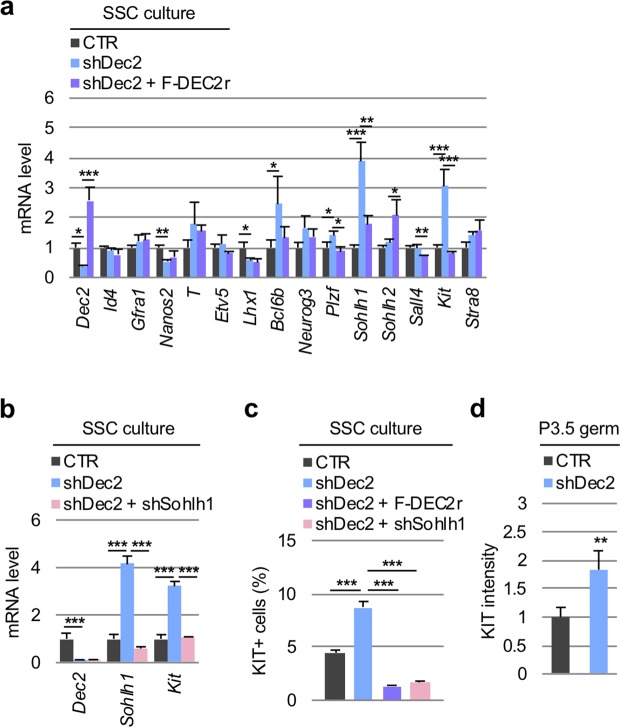
DEC2 transcriptionally suppresses *Sohlh1* and subsequently silences *Kit* expression both *in vitro* and *in vivo*. (**a**) Effects of *Dec2* knockdown (shDec2) and add-back (shDec2 + F-DEC2r) on the expression levels of selected marker genes in SSC culture. Five days after shRNA-containing lentivirus infection, cultured SSCs were harvested and subjected to RT-qPCR. (**b**) Effect of simultaneous knockdown of *Dec2* (shDec2) and *Sohlh1* (shSohlh1) on the expressions of *Dec2, Sohlh1*, and *Kit* mRNAs in SSC culture. In (**a**,**b**), the vertical axes indicated the relative mRNA expression levels (CTR = 1). Dunnett’s test was performed against shDec2 samples. (**c**) Effects of the combination of shDec2, F-DEC2r, and shSohlh1 on the number of KIT(+) cells in SSCs. Dunnett’s test against shDec2 samples was performed for statistical analysis. (**d**) Effect of shDec2 on KIT expression level in P3.5 germ cells. KIT intensity was measured by flow cytometry at three days after shDec2 lentivirus infection. The vertical axes indicated the relative KIT intensity (CTR = 1). Cell sorting scheme and raw flow cytometry data are shown in Fig. S12. Student’s t-test was performed for statistical analysis. All the experiments were performed by three biological replicates. Error bars indicated standard deviations. *p value < 0.05; **p value < 0.01; ***p value < 0.001.

Given that DEC2 is one of transcriptional repressors^37^, we next tested the molecular mechanism of DEC2 in maintaining undifferentiated spermatogonia through its transcriptional effect. Since we have observed transient and subtle expressions of differentiating spermatogonial markers *Sohlh1*, *Sohlh2*, and *Kit* at the beginning of the pseudotime (i.e. Cluster 1/quiescent gonocytes), and *Dec2* exhibited an anti-correlated expression pattern to these markers in this period (Figs S2c, S5, S6b), we hypothesized that DEC2 transcriptionally suppressed these differentiation drivers similar to its function in maintaining mesenchymal stem cells. To verify this hypothesis, RT-qPCR analysis for spermatogonial marker genes in *Dec2* knockdown cultured SSCs was performed. The result demonstrated that *Sohlh1* and *Kit* expressions were upregulated by *Dec2* knockdown and successfully cancelled by add-back of Flag-tagged DEC2 (Fig. 5a), suggesting the specific and suppressive effect of DEC2 on these genes. This result was also consistent with the inversely correlated response of *Dec2* and *Sohlh1/Kit* expressions to GDNF depletion (Fig. 3e). According to a previous report that SOHLH1 controls *Kit* expression during spermatogenesis^54^, we next examined the hierarchical dependency between *Dec2, Sohlh1*, and *Kit* in their transcriptional regulation. Strikingly, additional knockdown of *Sohlh1* resulted in cancellation of the *Kit* upregulation induced by *Dec2* knockdown (Fig. 5b). This finding strongly suggests that transcriptional suppression of *Kit* by DEC2 is a consequence of DEC2-induced transcriptional repression of *Sohlh1*. Consistently, KIT protein expression also increased *via* Dec2 knockdown and was restored by not only simultaneous overexpression of Flag-DEC2 but also Sohlh1 knockdown (Fig. 5c). Importantly, Dec2 knockdown also increased KIT expression in germ cells freshly isolated from P3.5 testes (Figs 5d, S12), further indicating the preventive role of DEC2 in germ cell differentiation in neonates.

**Figure 6:**
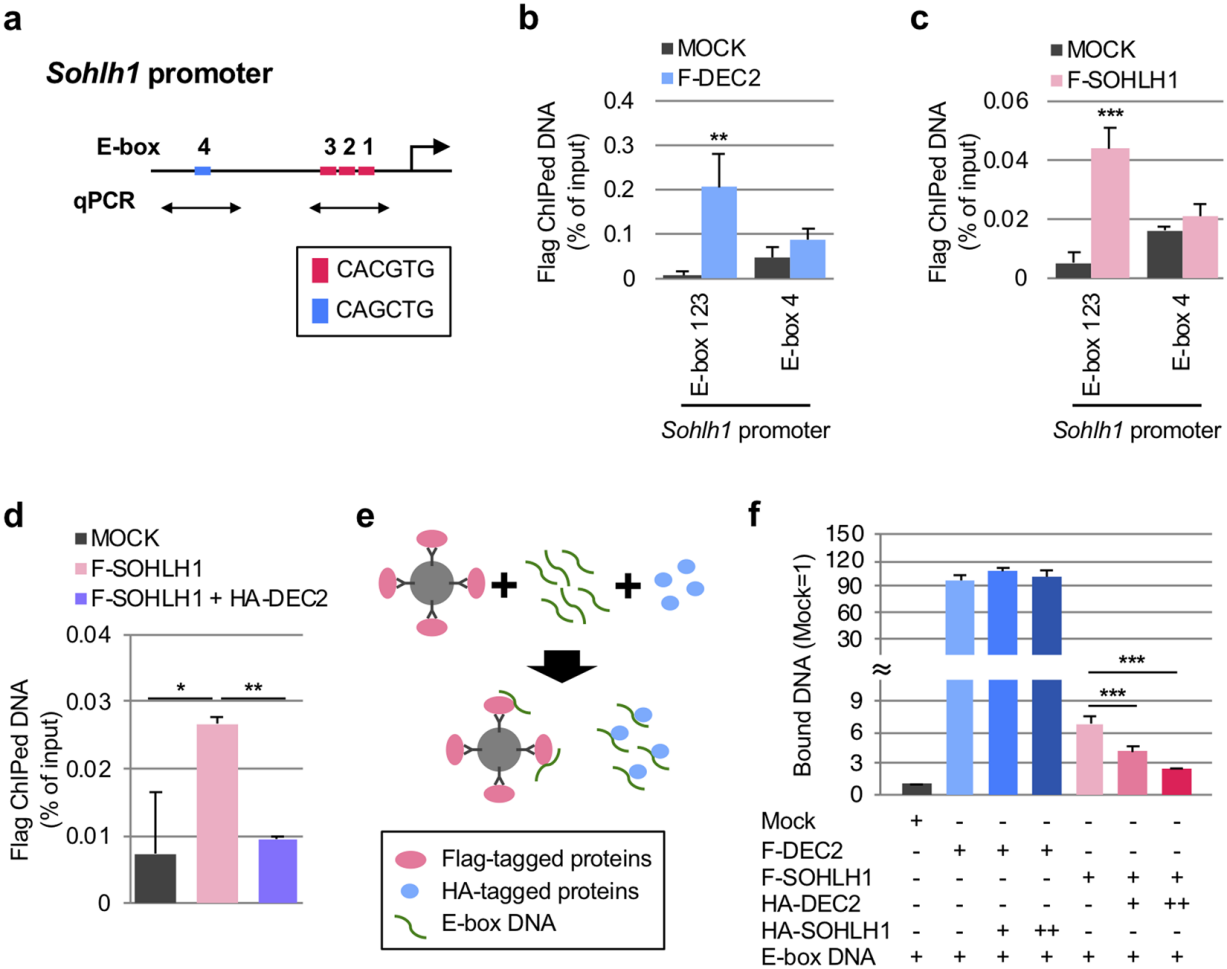
DEC2 competitively binds to E-box motifs on *Sohlh1* promoter with SOHLH1. (**a**) Scheme of *Sohlh1* promoter. E-box sequences and ChIP-qPCR primer sets are indicated. (**b,c**) ChIP-qPCR analysis demonstrating the binding of F-DEC2 (**b**) and F-SOHLH1 (**c**) to E-box 123 motifs of *Sohlh1* promoter. Student’s t-test was performed for statistical analysis. (**d**) ChIP-qPCR analysis demonstrating the inhibition of F-SOHLH1 binding to the E-box 123 motifs by HA-DEC2. Dunnett’s test against F-SOHLH1 samples was performed for statistical analysis. (**e**) Scheme of *in vitro* promoter binding assay (See materials and methods for details). (**f**) Competition assay of DEC2 and SOHLH1 for the binding of *Sohlh1* E-box motifs. Amounts of the DNA probes bound to Flag-tagged proteins were quantified by qPCR. Dunnett’s test was performed against the samples without HA-tagged proteins. All the experiments were performed by three biological replicates. Error bars indicated standard deviations. *p value < 0.05; **p value < 0.01; ***p value < 0.001.

Based on the previous finding that DEC2 represses transcription by binding to E-box motifs^37^, we next examined whether DEC2 was capable of targeting E-box motifs on the *Sohlh1* proximal promoter^30^ (three “CACGTG” E-box motifs, E-box 1, 2, and 3, −231 to −187 bp, Figs 6a, S14a). In the cultured SSCs expressing Flag-DEC2, ChIP-qPCR assay demonstrated that Flag-DEC2 was significantly enriched in the region containing E-box 123 (Figs 6b, S13a). the ChIP-qPCR assay also confirmed the self-association of Flag-SOHLH1 with the E-box 123 on its own promoter, as previously reported^30^ (Figs 6c, S13a). Because the binding of SOHLH1 to the *Sohlh1* promoter activates its own transcription^30^, these results indicated the possibility that DEC2 interferes with the binding of SOHLH1 to E-box 123 in a competitive manner. To test this, we performed ChIP-qPCR at E-box 123 when Flag-SOHLH1 and HA-DEC2 were simultaneously co-expressed (Fig. S13b). The result clearly demonstrated that HA-DEC2 inhibited the association of Flag-SOHLH1 with E-box 123 (Fig. 6d).

**Figure 7:**
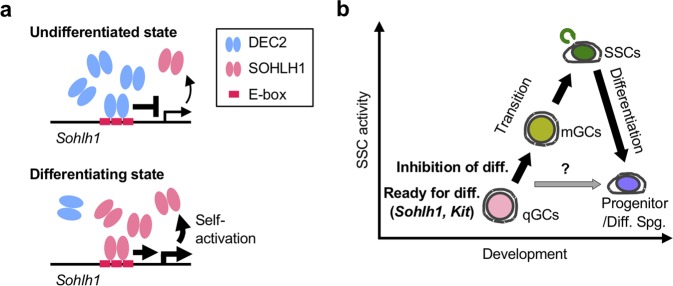
Proposed models of gonocyte-to-spermatogonia transition and regulation of *Sohlh1* transcription by DEC2 and SOHLH1. (**a**) Upper panel, competitive binding of DEC2 to the E-box motifs of *Sohlh1* promoter in undifferentiated state; lower panel, self-activation of *Sohlh1* promoter by SOHLH1 in differentiating state. (**b**) Processes of gonocyte-to-spermatogonia transition and spermatogonial differentiation. qGCs, quiescent gonocytes; mGCs, migratory/mitotic gonocytes; Spg., spermatogonia; diff., differentiation.

*In vitro* competition assay further demonstrated that Flag-DEC2 bound the E-box motif DNA (Figs. S14a–c), and that this binding was significantly altered when E-box motifs were mutated (Figs. S14a, c). Flag-SOHLH1 also bound to the E-box motif DNA with much less affinity, although similar amounts of Flag-DEC2 and Flag-SOHLH1 were applied to the reactions (Figs. S14b, c). Similar to the result of ChIP-qPCR assay, the addition of HA-DEC2 to the reaction significantly inhibited the association between Flag-SOHLH1 and E-box motif DNA, while the addition of HA-SOHLH1 to the reaction had no effects on the association between Flag-DEC2 and the E-box DNA (Figs 6e, f, S14d). Together with the ChIP-qPCR results, we concluded that DEC2 preferentially associated with the *Sohlh1* promoter and suppressed the transcriptional activation of *Sohlh1* (Fig. 7a).

## Discussion

The molecular basis of gonocyte-to-spermatogonia transition in neonatal testis has been an important and abstruse question due to the heterogeneous cell population occurring during this period. Recent scRNA-seq studies have begun to shed light on this issue^8–15^. In this study, we performed scRNA-seq of P1.5–5.5 male germ cells and focused on the temporal transition of gene expression profiles. This analysis allowed us to clarify not only the temporal expression dynamics of known spermatogonial factors but also the existence of the possible groups of TFs to regulate spermatogonial gene expressions (Modules 1 and 5) as well as the TFNs orchestrating gonocyte expression profiles (Modules 2, 3, and 4) (Fig. 2). For instance, *Rhox10* gene, which is required for establishing SSCs in neonatal testis^13,55^, was found in the early pseudotime network TFN Module 3 (Fig. 2a, Table S5), while the late pseudotime network Module 1 contained an undifferentiated spermatogonia marker gene *Plzf*, supporting the relevance of our analyses.

In contrast, although previous research has suggested the existence of different gonocyte subsets, some of which preferentially become SSCs, while others directly differentiate to type A spermatogonia^3,4^, we were unable to identify such distinct subsets similar to the observation in a recent report^12^. As discussed in that report, there could be several explanations such as insufficient number of cells and sequencing depth analyzed and the importance of post-transcriptional regulation. In addition, our study also recognized significant numbers of neonatal germ cells expressing Sertoli cell markers consistent with a previous observation^15^. Although substantial protein expressions of Sertoli cell markers were missed in these cells, a recent ATAC-seq study of human SSCs also demonstrated the significant enrichment of *Sox9* motif^8^, suggesting a possible involvement of *Sox9* in spermatogonia. Nevertheless, our study confirmed the usefulness of the scRNA-seq for systematic analyses of asynchronized neonatal germ cells.

Additionally, our analysis uncovered unexpected bimodal expressions of spermatogonial differentiation markers such as *Sohlh1* and *Kit* (Fig. S5) and further identified *Dec2* as a repressor of these differentiation genes in undifferentiated spermatogonia. *Dec2* was first identified as a paralogue of *Dec1*, which is involved in controlling cell proliferation and differentiation. Unlike the ubiquitous expression of *Dec1*, *Dec2* exhibits preferential expression in brain and muscles^33^. Both DEC1 and DEC2 were later found to repress CLCOK/BMAL1-induced transactivation of the mouse *Per1* promoter through direct protein-protein interaction with BMAL1 and/or competition for E-box elements^34^. More recently, the involvement of DEC2 in chondrocyte differentiation from mesenchymal stem cells^38–40^ as well as cancer cell progression^56–58^ has also been demonstrated. Strikingly, a recent scRNA-seq study also identified *Dec2* as one of the DEGs preferentially expressed in mouse spermatogonia^12^, while its role in germ cells has not been investigated. Experimentally, we demonstrated that DEC2 was enriched in GFRα1(+) early undifferentiated spermatogonia and its expression was regulated by the GDNF signaling pathway. Considering the fact that SOHLH1 was mainly detected in late undifferentiated spermatogonia onwards^20,21^, our demonstration of suppressed *Sohlh1* expression by DEC2 through competitive association with the *Sohlh1* promoter suggests that DEC2 maintains an undifferentiated state by preventing inadequate differentiation during neonatal testis development (Fig. 7b). To support this idea, we also demonstrated that *Dec2* knockdown in neonatal germ cells increased the expression of KIT, which is transactivated by SOHLH1 (Fig. 5d).

Regarding the competition between DEC2 and SOHLH1 on *Sohlh1* E-box motifs, our ChIP analysis suggested a higher affinity of DEC2 to the E-box motifs (Figs. 6b, c), which may ensure the effective repression of *Sohlh1* transcription. In fact, based on our *in vitro* promoter binding assay combined with semi-quantitative PCR, the affinity of DEC2 towards *Sohlh1* E-box motifs was significantly higher than that of SOHLH1. Interestingly, a recent study has demonstrated that DEC2 directly associates and suppresses the ligand-dependent transactivation of retinoid receptors (RXRs) including their heterodimers with retinoic acid receptors (RARs) or liver X receptors (LXRs) in HEK293 cells^59^. Since RA and expressions of RARs and RXRs are crucial inducers of germ cell differentiation^23^, the inhibition of RARs/RXRs might also be involved in an anti-differentiation effect of DEC2 in neonatal male germ cells.

It is also intriguing to ask how bHLH proteins coordinate in neonatal male germ cells, as many are expressed simultaneously and play crucial roles. bHLH proteins can be divided into six groups (A–F), depending on their target sequences (i.e. E-box motif)^28^. So far, *Id4* (group D), *Neurog3* (group A), and *Myc/Mycn* (group B) have been implicated in SSC self-renewal and differentiation^28,51,60–62^. *Dec2* belongs to group B, suggesting functional interactions with *Myc/Mycn*^28,63^. Group D factors compete with group A factors^28^, and the expressions of ID4 and NEUROG3 are mutually exclusive in undifferentiated spermatogonia^17,64^, suggesting an possibility of functional interplay in their transcriptional regulation. Further investigation regarding functional interactions among bHLH proteins will uncover elaborated cis- or trans-controls in gonocyte-to-spermatogonia transition and spermatogonial differentiation.

## Materials and Methods

See supplementary materials and methods for the full information and experimental details of single cell RNA-seq analysis and *in vitro* promoter binding assay. Information of antibodies and primer sets were listed in Table S7 and S8, respectively.

### Animals and ethics statement

For mouse experiments, C57BL/6, DBA/2, ICR, and BDF strains (75% C57BL/6 x 25% DBA/2 strain) (CLEA Japan, Tokyo, Japan) and H4V transgenic mouse (C57BL/6)^41^, Sox9-EGFP mouse^65^, Vas-RFP transgenic mouse was obtained from RIKEN Bioresource Reaearch Center (RBRC03449, Tsukuba, Japan), and *Dec2 (Sharp1)* knockout mouse^53^ lines were used for experiments. All the experimental procedures involving mice were approved by the Animal Experiment Ethics Committees at the Institute of Molecular and Cellular Biosciences, University of Tokyo (Exp # 2710, 2807, 2908). Experiments were performed in precise accordance with the manual provided by the Life Science Research Ethics and Safety Committee, University of Tokyo.

### Cells

For SSC cultures, Thy1^+^ germ cells were collected from DBA/2 and BDF mouse pups and maintained as described previously^66^. Briefly, the germ cells were cultured on mouse embryonic fibroblast (MEF) feeder cells in SSC culture medium, which consisted of basal culture medium for SSCs supplemented with Knockout Serum Replacement (10% for DBA/2 SSCs; 2% for BDF SSCs and testicular cells)(Gibco, Carlsbad, CA), 0.2% (w/v) BSA (MP Biochemicals, Santa Ana, CA), and 20 ng/ml human GDNF, unconjugated (Peprotech, Rocky Hill, NJ). While BDF strain cells were subjected to the transplantation experiments, DBA/2 were to the other experiments. For the experiments of GDNF depletion, the cultured DBA/2 strain SSCs were washed three times and cultured in GDNF-free SSC culture medium for the indicated periods. HEK293T cells were purchased from RIKEN Bioresource Research Center (RCB2202).

### Antibodies and Reagents

The antibodies used were listed in Table S7. The following reagents were used in the analyses: blasticidin S hydrochloride (029-18701, Wako Pure Chemical Industries, Ltd.), complete protease inhibitor cocktail (11697498001, Roche Applied Science, Basel, Switzerland), and DNase I (DN25, Sigma-Aldrich, St. Louis, MO).

### Plasmids

For production of recombinant DEC2 protein, *Dec2* (NM_024469.2) coding sequence fragments encompassing base pairs 763–1230 (amino acids 255–410 of DEC2 protein [NP_077789.1]) was inserted into pET15b-rrnBT1T2 with NdeI-BamHI digestion^67^. The resultant plasmid was named pET15b-Dec2. The lentiviral vectors (CS-RfA-EVBsd and CSII-EF1-IRES2-Bsd), the packaging plasmids (pCAG-HIVgp and pCMV-VSV-G-RSV-Rev), and a Gateway entry vector pENTR4-H1 were gifts from Dr. Hiroyuki Miyoshi (Riken, Tsukuba, Japan). For puromycin selection, Bsd sequence in CSII-EF1-IRES2-Bsd was replaced with puromycin-resistant gene (CSII-EF1-IRES2-puro). The shRNA target sequences for mCherry, mouse *Dec2* (NM_024469.2), and *Sohlh1* (NM_001001714.1) genes were as follows: mCherry-587 (GCGCCTACAACGTCAACATCA); *Dec2* #1 (GCAGTAGTCTTGGAATTAACT); *Dec2* #2 (GGACGAAGGAATCCCTCATTT); *Dec2* #3 (GGTTTCAAACCTGCGCCAAAG); *Sohlh1* (GGCTCTACTGCCTCAGTTTGA). The annealed DNA oligos of mCherry-587, *Dec2* #1, #2, #3, and *Sohlh1* were inserted into pENTR4-H1 at BglII-XbaI sites. The pENTER4 plasmids and CS-RfA-EVBsd were then recombined with LR clonase (Invitrogen, Carlsbad, CA). The resulting plasmids were named CSi-control, -shDec2 #1, #2, #3, and shSohlh1, respectively. The coding sequence of mouse *Dec2* and *Sohlh1* were amplified by PCR from mouse testis cDNA. The amplified fragments were cloned into CSII-EF1-IRES2-Bsd and CSII-EF1-IRES2-puro vectors with Flag-tag or HA-tag sequences. The resultant plasmid was named CSII-Flag-Dec2-Bsd, CSII-Flag-Sohlh1-Bsd, CSII-HA-Dec2-puro, and CSII-HA-Sohlh1-puro. CSII-Flag-Dec2-Bsd plasmid was mutated in cDNA sequence of *Dec2* (A261T, C264G, G267A, A270G, and A273G) to generate a silent mutant form resistant to shDec2 #1. The resultant plasmid was named CSII-Flag-Dec2r-Bsd.

### Single cell RNA-sequencing (scRNA-seq)

H4V testis tissues were dissociated with trypsin. According to the manufacturer’s instruction, the single cell suspensions were applied into C1 single cell library preparation system (Fluidigm, San Francisco, CA), and the scRNA-seq libraries were constructed using Nextera XT DNA Sample Preparation Kit (FC-131–1096, Illumina, San Diego, CA), followed by sequencing with HiSeq 2500 (Illumina). Bioimfomatical analyses were performed using the following software: STAR software^68^; seqtak software (https://github.com/lh3/seqtk); Singular Analysis Tool Set (Fluidigm); Monocle package (version 2.4.0, http://cole-trapnell-lab.github.io/Monocle-release/)^69^; WGCNA R package, Cytoscape (v3.2.1)^70^. Gene ontology analysis was carried out using DAVID bioinformatics resources^71^. Statistical analysis for DEGs between Clusters 1–6 was carried out by Steel-Dwass test.

### Mouse polyclonal anti-DEC2 antibody production

Recombinant mouse DEC2 protein was purified from *E. coli* (BL21 strain) transformed with pET15b-Dec2 using Ni-NTA affinity gel, followed by dialysis with PBS. The recombinant proteins were mixed with Adjuvant, Complete (Freund) (263810, BD, Franklin Lakes, NJ) for first time injection and Adjuvant, Incomplete (Freund) (263910, BD) for the residual injections. The mixtures of the recombinant proteins and adjuvants were subcutaneously injected into mice four times every two weeks. Two weeks after the fourth injection, whole blood was harvested, and the serum was subjected to the subsequent experiments.

### Western blotting

Tissue samples were harvested from C57BL/6 mice. For sample preparation, the cells were suspended with western blotting sample buffer and boiled for 5 min. The samples were separated by SDS-PAGE and transferred onto PVDF membranes (Merck Millipore, Darmstadt, Germany). After blocking with 5% skim milk in TBST, the membranes were incubated with primary antibodies followed by secondary antibodies. Positive signals were detected using ImageQuant LAS 4000 (GE Healthcare, Chicago, IL). As secondary antibodies, Mouse TrueBlot ULTRA: Anti-Mouse Ig HRP was used for detection of immunoprecipitated samples, while Goat anti-Mouse IgG (H + L) Secondary Antibody-HRP and Goat anti-Rat IgG (H + L) Secondary Antibody-HRP were for regular western blotting.

### Immunohistochemistry

Testes were harvested, fixed with 4% paraformaldehyde in PBS O/N at 4 °C, and then embedded in O.C.T Compound (4583, Sakura Finetek Japan, Tokyo, Japan). Frozen sections (10 μm in thickness) were incubated with following blocking buffers for 1 h at RT: blocking buffer 1 (TBST containing 5% goat normal serum) for DEC2 staining; blocking buffer 2 (PBS containing 2% BSA and 0.2% Triton X-100) for GFRα1 and double staining of DEC2 and GFRα1, respectively. The sections were then incubated with primary antibodies diluted in the same buffers at the blocking step for 1 h at RT or O/N at 4 °C. After wash with TBST, the sections were incubated with secondary antibodies for 1 h at RT. Nuclear staining was carried out with Hoechst 33342. Images were obtained by confocal microscopy (FV3000, OLYMPUS, Tokyo, Japan). In double staining of DEC2 and GFRα1, DEC2 intensities in germ cells, which were GENA/TRA-98 positive, were quantified using ImageJ software (https://imagej.nih.gov/ij/). The intensities that were lower than the first quartile, between the first and third quartiles, and higher than the third quartile were designated as “low”, “mid”, and “high” populations, respectively. Then, proportions of low, mid, and high populations in GFRα1(+) and GFRα1(−) germ cells were calculated. In wild type and knockout testis sections, the numbers of GFRα1(+) germ cells were counted.

### Cell sorting

P7.5 testes were harvested from H4V mice and trypsinized to prepare the single cells. The single cell suspensions were incubated with anti-GFRα1. After three-time wash, the cells were then incubated with anti-mouse IgG Alexa568 and anti-KIT-APC antibodies. The stained cells were subjected into cell sorting with BD FACSAria III (BD biosciences, Franklin Lakes, NJ).

### Reverse transcription-quantitative PCR (RT-qPCR)

Cultured SSC samples were trypsinized and purified using a feeder cell removal kit (Miltenyi Biotech, Bergisch Gladbach, Germany). Total RNA was then extracted with NucleoSpin RNA II kit (Macherey-Nagel, Düren, German), and then reverse transcription reaction was performed with the PrimeScript RT reagent Kit (TaKaRa BIO, Otsu, Japan) according to the manufacturer’s protocols. qPCR samples were prepared with the KAPA SYBR FAST ABI Prism 2X qPCR Master Mix (Kapa Biosystems, Wilmington, MA), followed by the qPCR reactions with ABI StepOne (Applied Biosystems, Foster City, CA). The results were normalized to the values obtained for mouse ribosomal 18S mRNA.

### Lentivirus infection

Lentiviral vectors were co-transfected with the packaging plasmids into HEK293T cells. After incubation, the supernatants were collected twice at day 2 and 3, passed through a 0.45-μm filter, and centrifuged at 8,000 × g for 14 h at 4 °C. The virus pellets were suspended in SSC culture medium and mixed with the single cell suspensions of cultured SSCs. The multiplicity of infection (M. O. I) was in the range from 100 to 200 for the knockdown experiments and 10 for the stable overexpression. After incubation for 3 h, the cells were washed with PBS and seeded onto MEF feeder cells. For establishment of stably expressing SSC lines, blasticidin S (10 μg/ml) and/or puromycin (0.3 μg/ml) were treated for six or seven days. Images of the cultured cells were obtained by epi-fluorescence microscopy (IX73, Olympus, Tokyo, Japan).

### Flow cytometry

For the confirmation of knockdown virus infection, the cultured SSCs were trypsinized and harvested at five days post infection (dpi). The single cell suspensions were treated with 1 μg/ml 7-Aminoactinomycin D (7-AAD, A-1310, ThermoFisher, Waltham, MA) for 30 min at RT. Venus-positive and 7-AAD-negative signals corresponded to the infected and viable cells, respectively. For surface staining, the single cell suspensions were incubated with KIT-APC for 30 min on ice and then washed three times with PBS containing 1% BSA. For intracellular staining, the single cell suspensions were fixed with 4% PFA-PBS for 20 min at RT. After washed three times with PBS containing 0.05% Tween-20 and 1% BSA (1% BSA-PBST), the cells were permeabilized with PBS containing 0.1% Triton X-100 for 30 min at RT. Anti-GENA/TRA98 (×2,500) and anti-GFRα1 (AF560, ×2,500) were treated for 1 h at RT. For secondary antibody treatment, PE anti-rat IgG2a antibody, F(ab’)2-Goat anti-Rabbit IgG (H + L) Secondary Antibody, and Alexa Fluor 647 conjugate (A-21246) were used. The samples were analyzed with flow cytometer (BD Accuri™ C6 flow cytometer, BD) to detect Venus and 7-AAD signals. Analysis was performed with Flowjo software (ver 7.6.5, Treestar, Ashland, OR).

### SSC transplantation

Transplantaion experiment was carried out as described previously with several modifications^41^. Four- to five-week-old *W/W*^*v*^ germ cell-depleted mice (*WBB6F1/Kit-KitW/KitW-v/Slc*, Japan SLC Inc., Hamamatsu, Japan) were used as recipients. Cultured BDF strain SSCs were infected with control and shDec2 #1 lentiviruses. At five days post infection (dpi), the cells were trypsinized immediately prior to transplantation and suspended with SSC culture medium containing 0.2 mg/ml DNase I to prepare single cell suspensions. 5 × 10^4^ (5 μl) of the cells were then injected into recipient testes (n = 4 or 5 per group). At two months after transplantation, the testes were removed from recipient mice, and the numbers of the colonies derived from engrafted SSCs were counted. A colony was defined as a contiguous Venus-positive region.

### Knockdown experiments of testicular cells

After P3.5 testes were harvested from ICR mice and trypsinized, the single cell suspensions were incubated with control or shDec2 #1 lentiviruses (MOI = 10–20) for 3 h. The cells were washed with PBS and suspended with SSC culture medium. The cells were seeded onto gelatin-coated 6-well culture plates (1 × 10^6^/well). At 3 dpi, the cells were harvested and subjected to intracellular staining.

### Chromatin immunoprecipitation (ChIP) assay

Cultured SSCs were suspended with 1% FBS-PBS at 2 × 10^6^/ml and cross-linked for 10 min at RT by adding the equal volume of 1% FBS-PBS containing 2% formaldehyde (1% final concentration). After the addition of glycine, the cells were washed twice with 1% FBS-PBS and then lysed in the lysis buffer consisting of 20 mM Tris-HCl (pH 7.4), 150 mM NaCl, 1 mM EDTA, 0.5 mM EGTA, 1% triton X-100, 0.1% DOC, and complete protease inhibitor cocktail, followed by centrifugation at 2,300 × g for 5 min. The pellets were suspended with 20 μl of SDS buffer consisting of 50 mM Tris-HCl (pH 7.4), 1 mM EDTA, and 1% SDS, incubated at RT for 30 min, and diluted by adding 80 μl of the lysis buffer, followed by sonication. After centrifugation at 20,000 × g for 10 min, the supernatants were subjected to immunoprecipitation with anti-FLAG antibody. The immunoprecipitated samples were washed three times with the lysis buffer and once with TE buffer consisting of 50 mM Tris-HCl (pH 8.0) and 10 mM EDTA. Elution was carried out with TE buffer containing 1% SDS for 20 min at 65 °C, and the eluates were de-crosslinked O/N at 65 °C. After RNase A and Proteinase K treatment, the DNA fragments were purified with AMPure XP (1.8×, A63880, Beckman Coulter, Brea, CA) and quantified by qPCR.

### *In vitro* promoter binding assay

For preparation of the DNA probe, the *Sohlh1* promoter sequence of −265 to −76 base pair (bp) was amplified by PCR. Flag- and HA-tagged proteins were purified from HEK293T cells. Flag-bead suspensions and the DNA probes were mixed and rotated for 1 h at 4 °C. After wash, the bound DNA probes were amplified by quantitative PCR (qPCR). For specific detection, the primer set of *Sohlh1* ChIP E-box 123 (-251 to -166 bp of *Sohlh1* promoter) was applied. The competition assay was performed by the addition of HA-tagged proteins into the mixture of the Flag-bead suspension and the DNA probe.

## Supplementary information

Supplementary information

Supplementary tables

## Data Availability

The GEO accession number for single cell RNA-seq data reported in this paper is PRJNA524391.
