# Supplementary material for: Single cell RNA-sequencing identified Dec2 as a suppressive factor for spermatogonial differentiation by inhibiting Sohlh1 expression
Source: Sci Rep. 2019 Apr 15;9:6063. doi: 10.1038/s41598-019-42578-z (PMC6465314; doi:10.1038/s41598-019-42578-z)
Supplement: Supplementary file 1 — Supplementary information [file 41598_2019_42578_MOESM1_ESM.pdf]

Single cell RNA-sequencing identified *Dec2* as a suppressive factor for spermatogonial differentiation by inhibiting *Sohlh1* expression

Yoshinori Makino[1], Niels H. Jensen[2], Naoko Yokota[3], Moritz J. Rossner[2], Haruhiko Akiyama[4], Katsuhiko Shirahige[3], & Yuki Okada[1*]

[1]Laboratory of Pathology and Development, Institute for Quantitative Biosciences, The University of Tokyo, 1-1-1 Yayoi, Bunkyo-ku, Tokyo 113-0032, Japan.

[2]Molecular and Behavioral Neurobiology, Department of Psychiatry and Psychotherapy, University Hospital, LMU Munich, Germany.

[3]Laboratory of Genome Structure and Function, the Institute for Quantitative Biosciences, University of Tokyo, 1-1-1 Yayoi, Bunkyo, Tokyo 113-0032, Japan.

[4]Department of Orthopaedic Surgery, Gifu University Graduate School of Medicine, Yanagido 1-1, Gifu, 501-1194, Japan.

Supplementary information

Supplementary Materials and Methods

Supplementary table legends

Supplementary figures

Raw images

Supplementary Materials and Methods

Single cell RNA-sequencing (scRNA-seq)

After the tunica was removed, the H4V testis tissues were dissociated with trypsin. According to the manufacturer's instruction, the single cell suspensions were applied into C1 single cell library preparation system (Fluidigm, San Francisco, CA) to construct scRNA-seq libraries. Briefly, cDNA libraries were generated from single cells captured by the Fluidigm C1 platform. The cDNAs were converted into sequence libraries using Nextera XT DNA Sample Preparation Kit (FC-131–1096, Illumina, San Diego, CA) and sequenced with HiSeq2500 (Illumina). After the quality check and trimming, the read mapping and the calculation of gene counts and TPMs were carried out using STAR software[1]. One million reads were randomly extracted from each sample using seqtak software (https://github.com/lh3/seqtk). The sequence data were analyzed by Singular Analysis Tool Set (Fluidigm) to exclude the samples showing aberrant patterns of gene expression profiles. The visualization and calculation for characterizing the samples were carried out by Monocle package (version 2.4.0, http://cole-trapnell-lab.github.io/Monocle-release/)[2]. Gene counts were applied to Monocle analysis. Expressed genes were defined when their expression levels were more than 1 in at least 10% of the samples. Negative binomial distribution was chosen. The parameters of t-SNE were as follows: max_components = 2; norm_method = "log"; num_dim = 10; scale = TRUE. In the clustering analysis, the value of rho_threshold was 2; that of delta_threshold was 4. The step of calculation of trajectory and pseudotime was performed by DEGs between Clusters 1–6 at, the parameters as follows: max_components = 2 ; norm_method = "log"; num_dim = 10; scale = TRUE. Pseudotime DEGs were identified in Clusters 1–3 samples. Gene ontology analysis was carried out using DAVID bioinformatics resources[3]. Same as Pseudotime DEG identification, only Clusters 1–3 samples were applied into Weighted gene co-expression network analysis (WGCNA) performed by WGCNA R package with the parameter as follows: power = 5; TOMType = "unsigned"; minModuleSize = 10; deepSplit = 2 [4]. The network

modules calculated by WGCNA were visualized by Cytoscape (v3.2.1)[5]. The genes contained in the detected network modules and their correlations (threshold = 0.001) were transferred to Cytoscape.

In vitro promoter binding assay

For preparation of the DNA probe, the Sohlh1 promoter sequence of -265 to -76 bp was amplified from mouse genomic DNA by nested PCRs: 1st PCR (-1,000 to +53 bp of Sohlh1 promoter) and 2nd PCR (-265 to -76 bp of Sohlh1 promoter). The PCR products were purified with MonoFas DNA Purification Kit I (5010-21530, GL Sciences, Tokyo, Japan). The DNA probe was mutated at all three E-box motifs (CACGTG to CAATTG, Fig. S14A). Sequences of both wildtype and mutants were verified. For preparation of Mock, Flag-tagged, and HA-tagged proteins, the plasmids of CSII-EF1-IRES2-Bsd (Mock), CSII-Flag-Dec2-Bsd (F-Dec2), CSII-Flag-Sohlh1-Bsd (F-Sohlh1), CSII-HA-Dec2-puro (HA-Dec2), and CSII-HA-Sohlh1-puro (HA-Sohlh1) were transfected to HEK293T cells in 6-cm dishes. Two days after transfection, the cells were lysed with the lysis buffer (See ChIP assay in Materials and Methods) and placed for 15 min on ice. The samples were centrifuged for 10 min at 4°C, and the supernatants were subjected to immunoprecipitation. The protein G beads conjugated with anti-Flag or anti-HA antibody were mixed with supernatants and rotated for O/N at 4°C. After washed three times with the lysis buffer, The Flag beads were suspended with 100 μl (MOCKk and F-SOHLH1) or 200 μl (F-DEC2) of the lysis buffer. The HA-beads were further washed once with TBST, followed by the elution of HA-tagged proteins with 100 μl of TBST containing 0.1 mg/ml 3×HA peptides. 1 μl of the Flag-bead suspensions, 3 μl of the lysis buffer, and 46 μl 1% BSA-PBST containing 0.1 ng of the DNA probes were mixed and rotated for 1h at 4°C. The beads were washed three times with 1% BSA-PBST to remove the unbound DNA probes and suspended with 10 μl of 1% BSA-PBST. 1 μl of the beads were applied to qPCR to quantify the amounts of bound DNA probes. For specific detection, the primer set of Sohlh1 ChIP E-box 123 (-251 to -166 bp of Sohlh1 promoter), which was distinct from the primer sets to amplify the

DNA probe, was used. The competition assay was performed by the addition of 2 μl (+) or 10 μl (++) HA-tagged proteins into the mixture of the Flag beads and the DNA probes. 10 μl of the Flag bead suspensions and 5 μl the HA-tagged proteins were subjected to western blotting to confirm the amounts and molecular weights. The antibodies and primer sets are listed in Table S1 and S2, respectively.

References

1 Dobin, A. *et al.* STAR: ultrafast universal RNA-seq aligner. *Bioinformatics* **29**, 15-21; 10.1093/bioinformatics/bts635 (2013).

2 Trapnell, C. *et al.* The dynamics and regulators of cell fate decisions are revealed by pseudotemporal ordering of single cells. *Nature Biotechnology* **32**, 381-U251; 10.1038/Nbt.2859 (2014).

3 Huang da, W., Sherman, B. T. & Lempicki, R. A. Systematic and integrative analysis of large gene lists using DAVID bioinformatics resources. *Nat Protoc* **4**, 44-57; 10.1038/nprot.2008.211 (2009).

4 Langfelder, P. & Horvath, S. WGCNA: an R package for weighted correlation network analysis. *BMC Bioinformatics* **9**, 559; 10.1186/1471-2105-9-559 (2008).

5 Busada, J. T. *et al.* Retinoic acid regulates Kit translation during spermatogonial differentiation in the mouse. *Dev Biol* **397**, 140-149; 10.1016/j.ydbio.2014.10.020 (2015).

Supplementary table legends

Supplementary table S1. Gene expression (TPM) matrix of all the expressed genes.

Supplementary table S2. Pseudotime DEGs (*q* value < 0.05).

Supplementary table S3. Gene ontologies enriched in GG1 to GG4 gene groups.

Supplementary table S4. Statistical analysis of marker genes.

Supplementary table S5. TFN Modules and statistical analysis of the pseudotime DEG transcription factors.

Supplementary table S6. Gene ontologies enriched in TFN Modules 1/5 and 2/3/4.

Supplementary table S7. Antibody list.

Supplementary table S8. Primer set list.

a

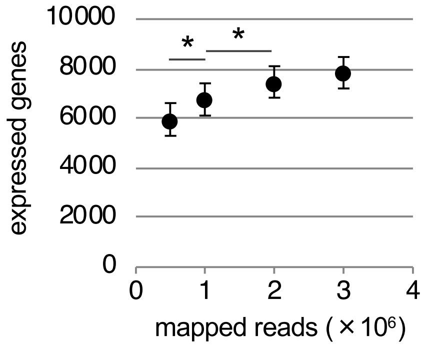

b

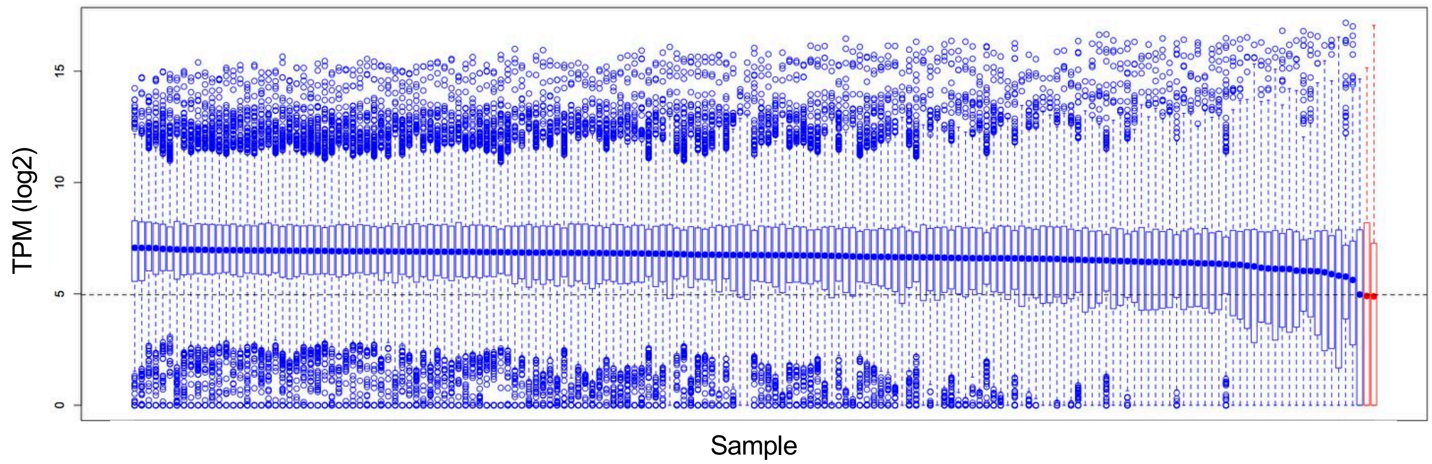

Supplementary figure S1 (related to Fig. 1). Primary quality validation of scRNA-seq data. (a) Correlation between the numbers of mapped reads and the numbers of expressed genes. The samples, the mapped reads of which were more than three million were applied to calculation. **p* value < 0.05. (b) Distribution of TPMs in each sample. The samples with abnormal distributions were indicated in red.

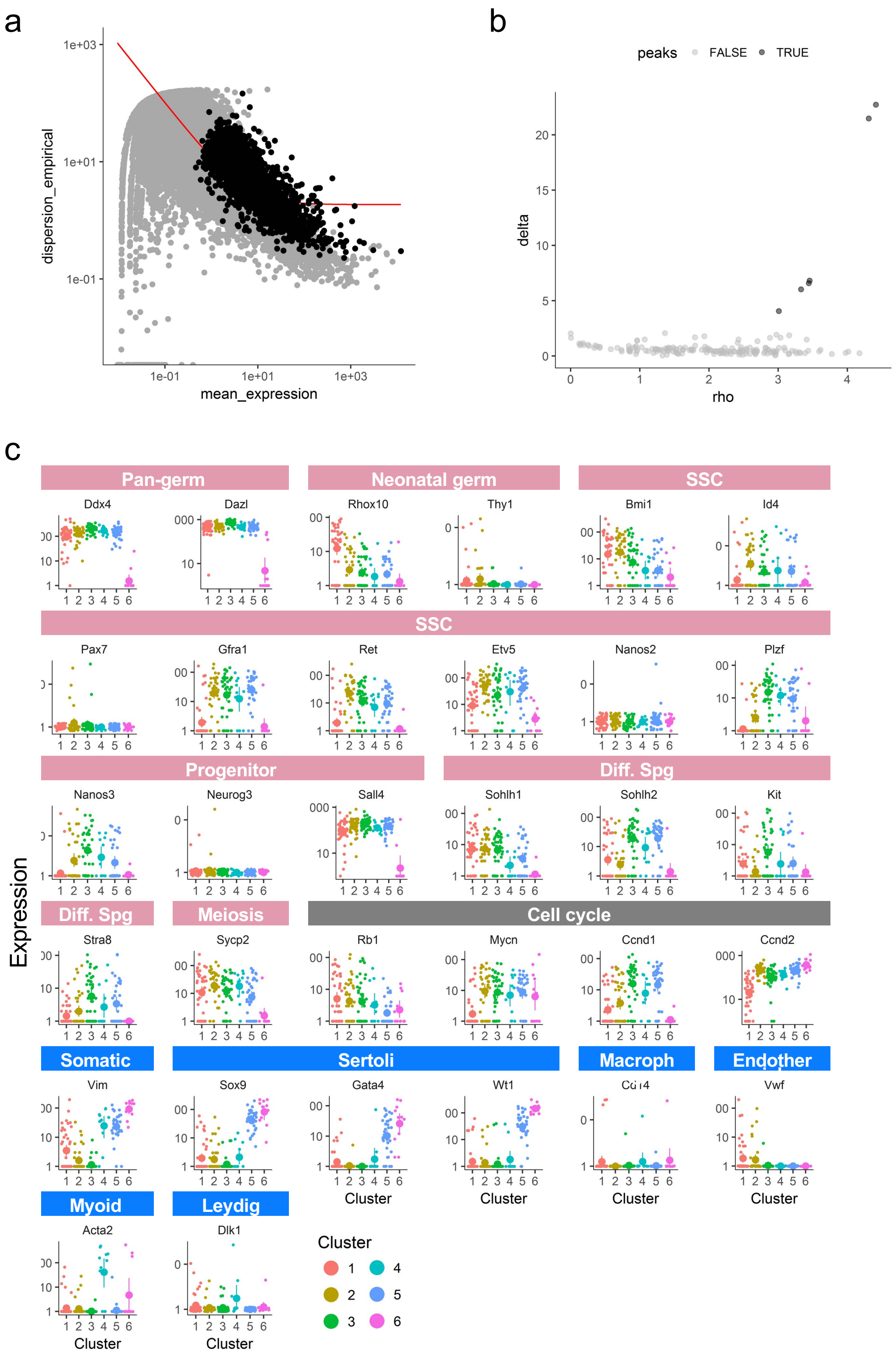

Supplementary figure S2 (related to Fig. 1b). Clustering analysis using all the expressed genes identified six subpopulations.

(a) Plot of mean expressions and dispersions of all the expressed genes. Differentially expressed genes between Clusters 1–6, which were applied into the reconstruction of the trajectory shown in Figure 1d, were denoted in black dots. (b) The decision plot to check the rho and delta values for clustering of the cell samples. Note that six TRUE peaks were indicated corresponding to the composition of six clusters (Clusters 1–6). (c) Jitter plots of marker gene expressions. Clusters 1–6 were depicted in colors as indicated. Properties of each marker gene are as indicated.

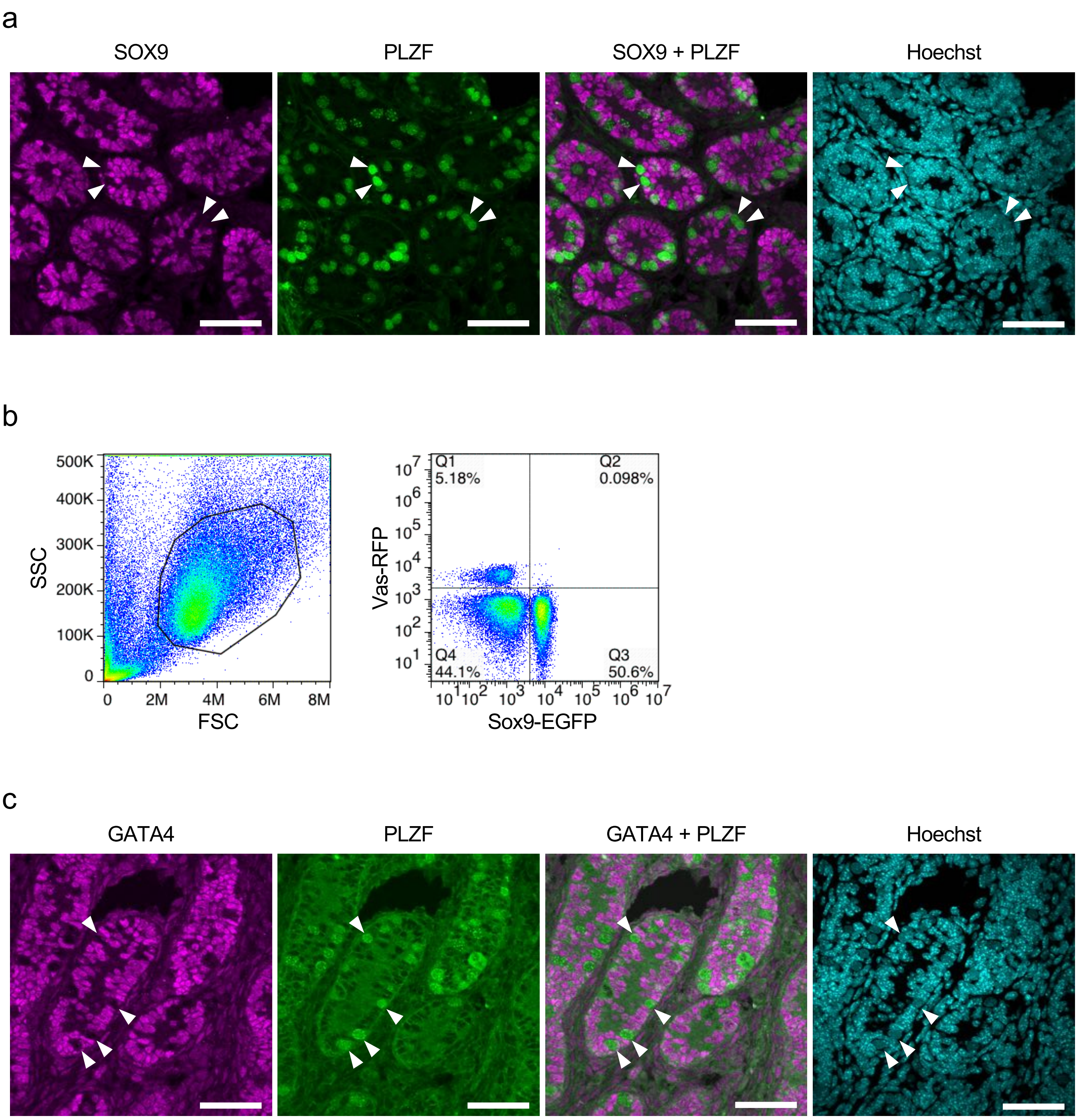

Supplementary figure S3 (related to Fig. 1c). Unsuccessful detection of SOX9 and GATA4 proteins in neonatal testis

(a) Immunohistochemical analysis of SOX9 protein expression in P5.5 neonatal testis. White arrowheads indicate germ cells. PLZF, an undifferentiated spermatogonial marker. Scale, 50 μm. (b) Flow cytometric analysis of Sox9-EGFP-expressing testis. The P4.5 testicular cells gated by FSC and SSC parameters (left panel) were shown in the right panel. Sox9-EGFP represented the transcript level of *Sox9*. Vas-RFP(+) cells corresponded to germ cells. (c) Immunohistochemical analysis of GATA4 protein expression in P5.5 neonatal testis. White arrowheads indicate germ cells. PLZF, an undifferentiated spermatogonial marker. Scale, 50 μm.

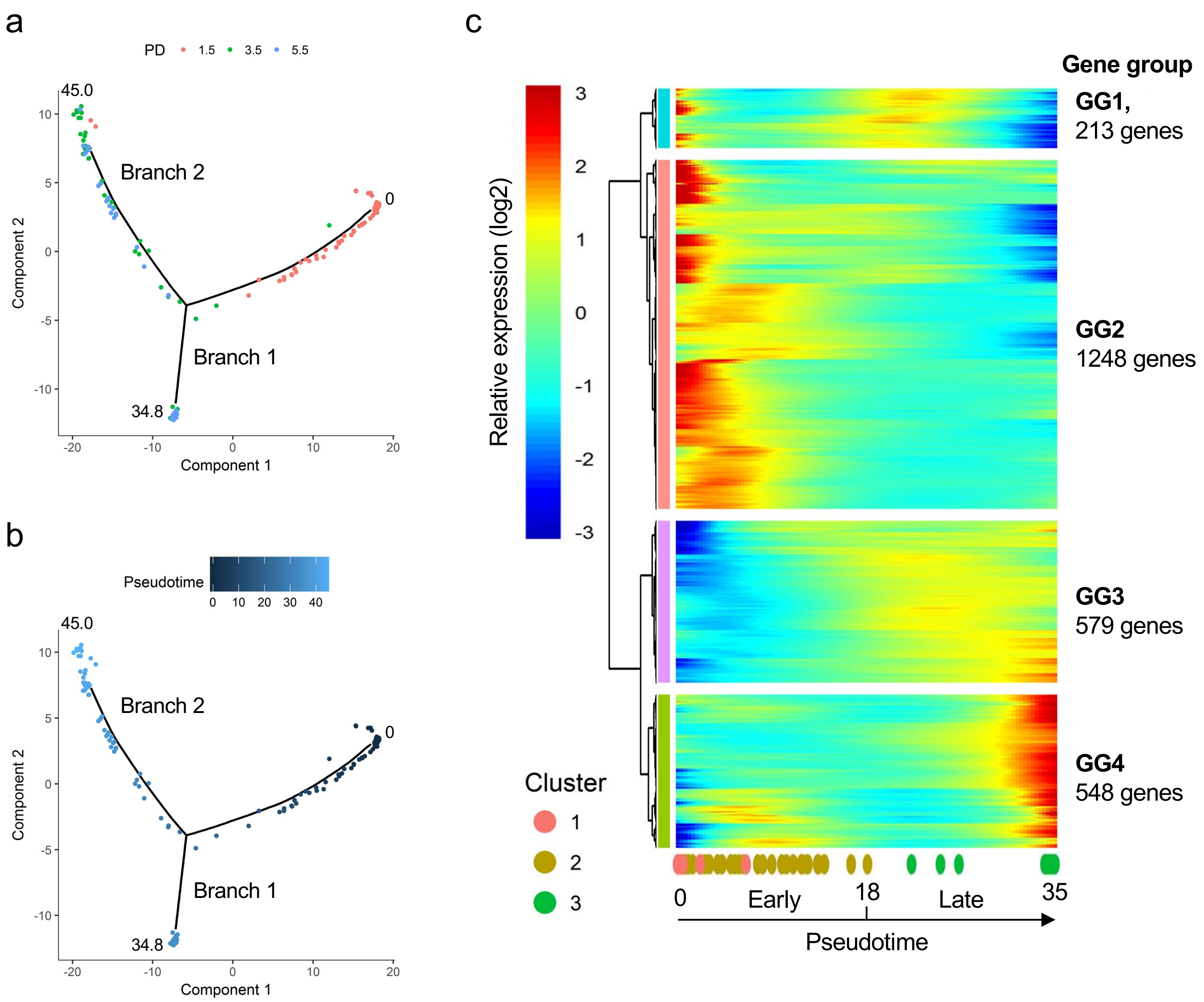

Supplementary figure S4 (related to Figs. 1d, 1e). Trajectory and pseudotime analyses. (a, b) Trajectory plots projecting the postnatal days (PD) (a) and peseudotime (b) instead of cell clusters shown in Fig. 1d. The earlier and later pseudotimes were denoted in darker and brighter blue colors, respectively. (c) Heatmap of pseudotime DEGs shown in Fig. 1e. Colors and terms correspond to those in Fig. 1e.

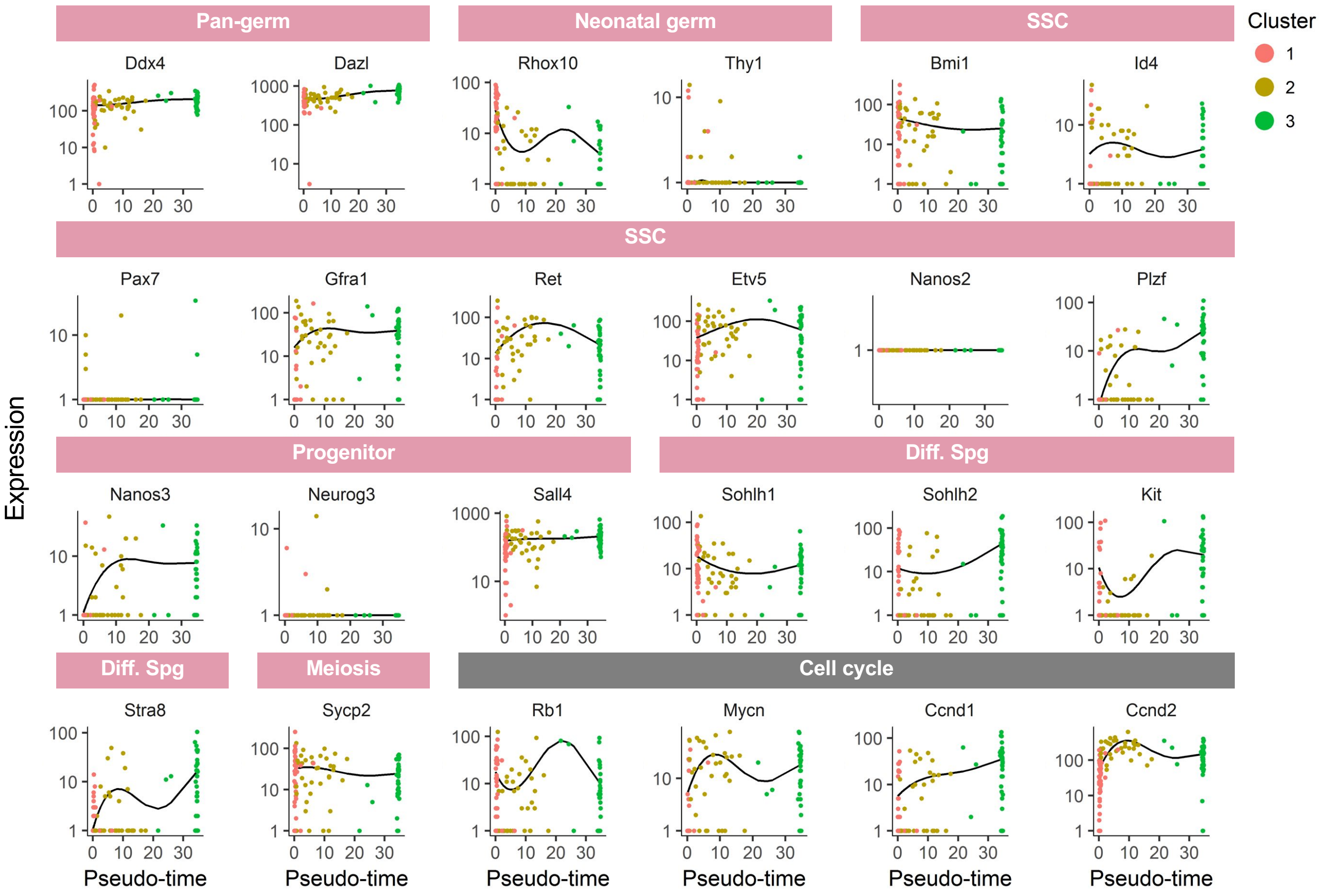

Supplementary figure S5 (related to Figs. 1d, 1e). Pseudotime expression profiles of representative germ cell marker genes.

Clusters 1–3 were depicted in colors as indicated. Black lines indicate the fitted curves of their expression levels. Properties of each marker are as indicated.

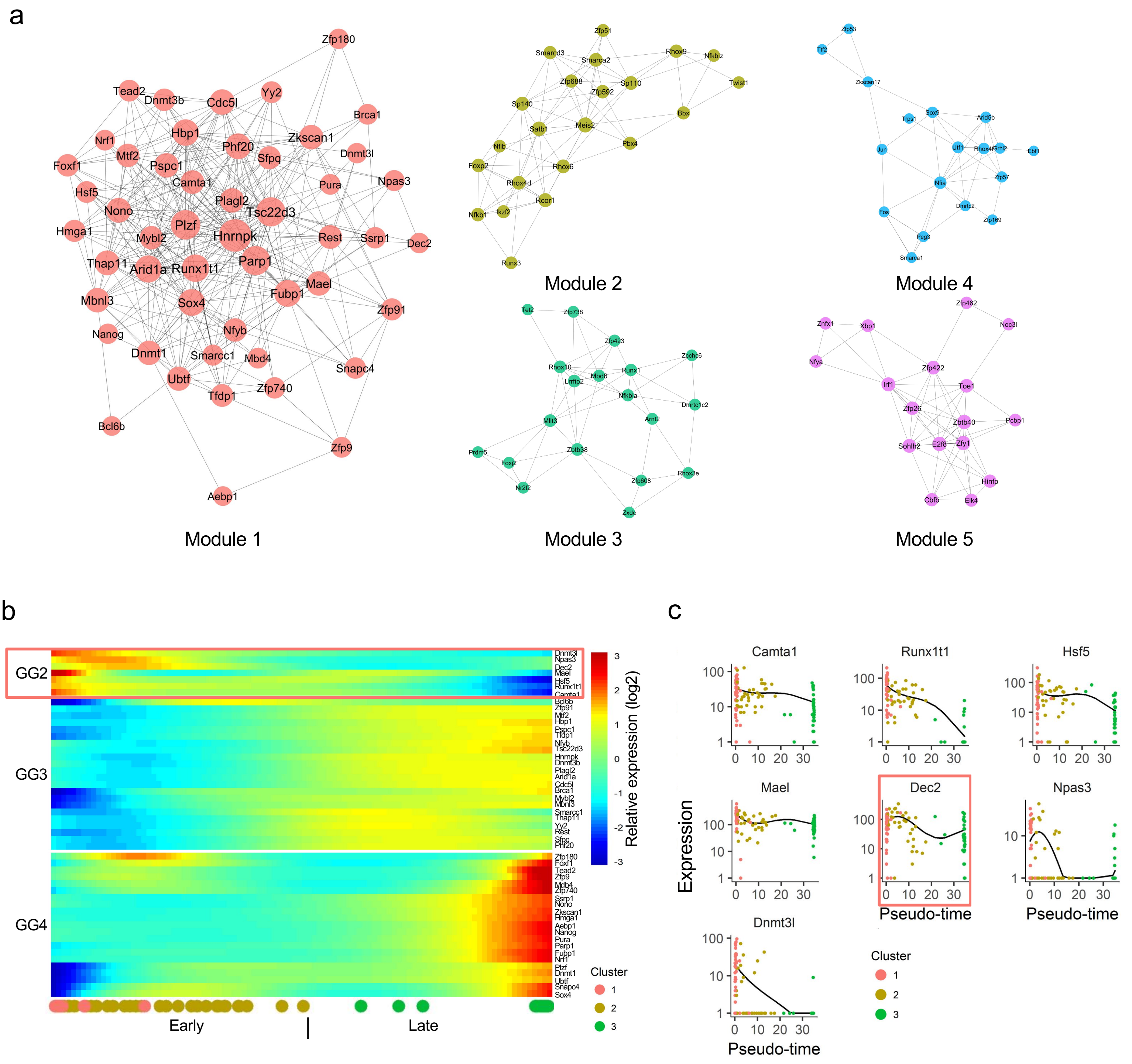

Supplementary figure S6 (related to Figs. 2). WGCNA identifies transcription factor network (TFN) modules and Module 1 as a potential spermatogonial TFN module.
(a) TFN modules shown in Fig. 2a. Each circle represents each gene, and lines between two circles indicate network connections (topological overlap > 0.001) between them. (b) Heatmap of pseudotime expression dynamics of Module 1 TFs shown in (a). Pseudotime attributions of Clusters 1–3 cells are depicted in colors as indicated at the lower side of the heatmap. (c) Pseudotime expression profiles of GG2 early TFs in Module 1. Black lines indicate the fitted curves of their expression levels. Clusters 1–3 are depicted in colors as indicated.

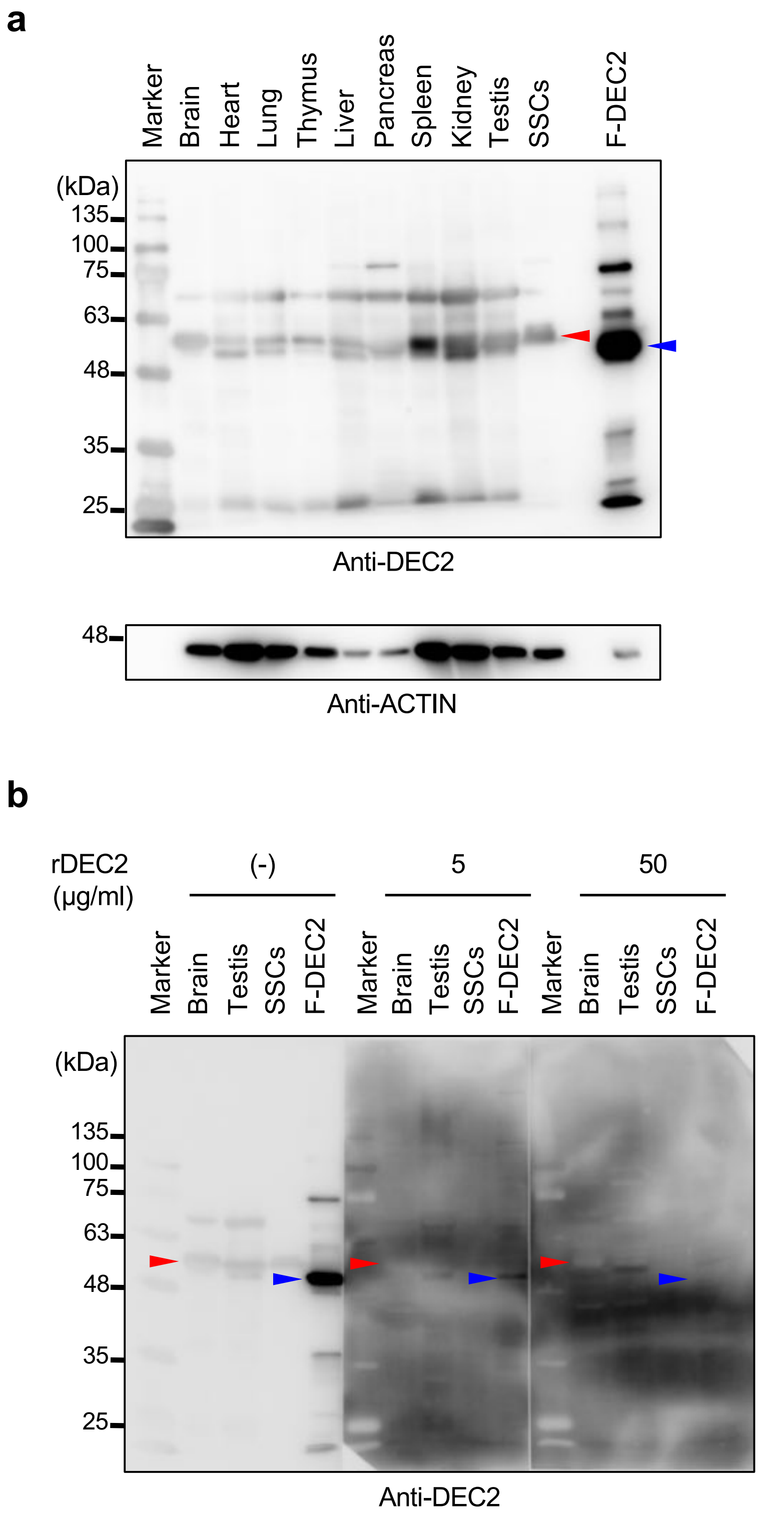

Supplementary figure S7 (related to Figs. 3a–c). Validation of newly generated anti-DEC2 antibody by western blotting.

(a) Detection of endogenously expressed DEC2 protein in various mouse tissues by Western blotting using anti-DEC2 antiserum. SSCs, cultured SSCs; F-DEC2, Flag-DEC2 overexpressed in HEK293T cells. In several tissues, two bands were detected: the lower band (~50 kDa), blue arrowhead; the higher band (~55 kDa), red arrowhead. ACTIN was provided as loading control.

(b) Absorption test of anti-DEC2 antiserum using recombinant DEC2 protein (rDEC2). Pre-mixtures of anti-DEC2 antiserum and the recombinant proteins (5 or 50 µg/ml) were applied to western blot analysis.

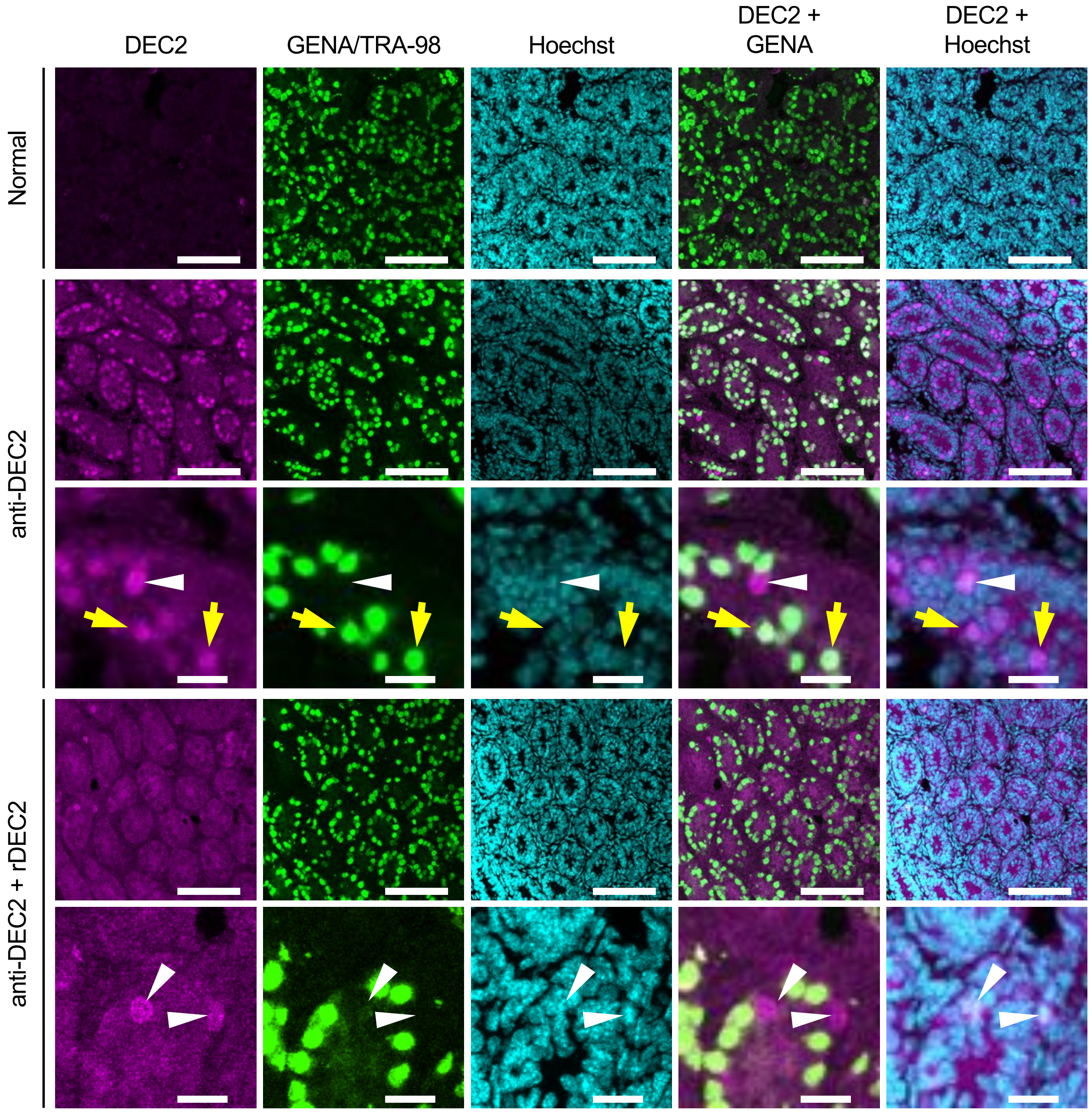

Supplementary figure S8 (related to Figs. 3a–c). Validation of newly generated anti-DEC2 antibody by immunohistochemistry.
P5.5 testis was subjected to immunohistochemistry using normal mouse serum (normal) and anti-DEC2 antiserum (anti-DEC2), respectively. GENA/TRA-98, a germ cell marker. Recombinant DEC2 protein (rDEC2) was used for absorption of anti-DEC2 antiserum. Yellow arrows and white arrowheads indicated DEC2 signals in germ cells and non-specific signals, respectively. Scales, 50 μm (the top, second, and fourth panels) and 20 μm (the third and bottom panels).

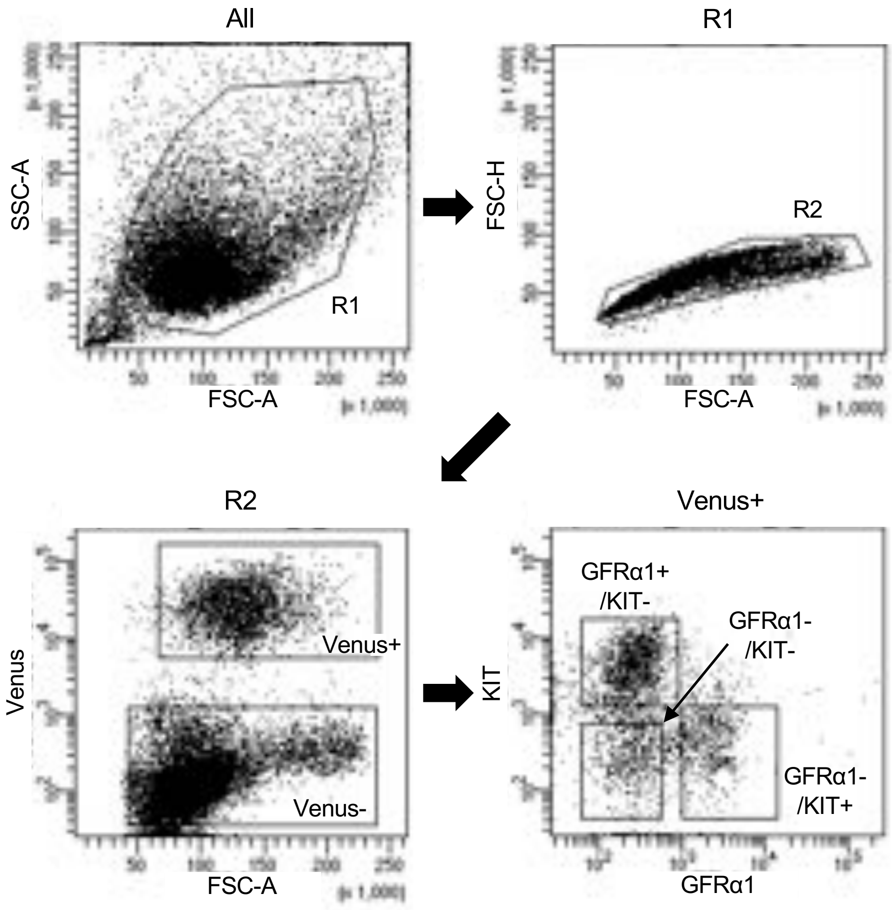

Supplementary figure S9 (related to Fig. 3d). FACS sorting scheme from H4-Venus testicular cells.

H4-Venus(-), H4-Venus(+)/GFRA1(+)/KIT(-), H4-Venus(+)/GFRA1(-)/KIT(-), and H4-Venus(+)/GFRA1(-)/KIT(+) populations were isolated from P7.5 H4-Venus testicular cells.

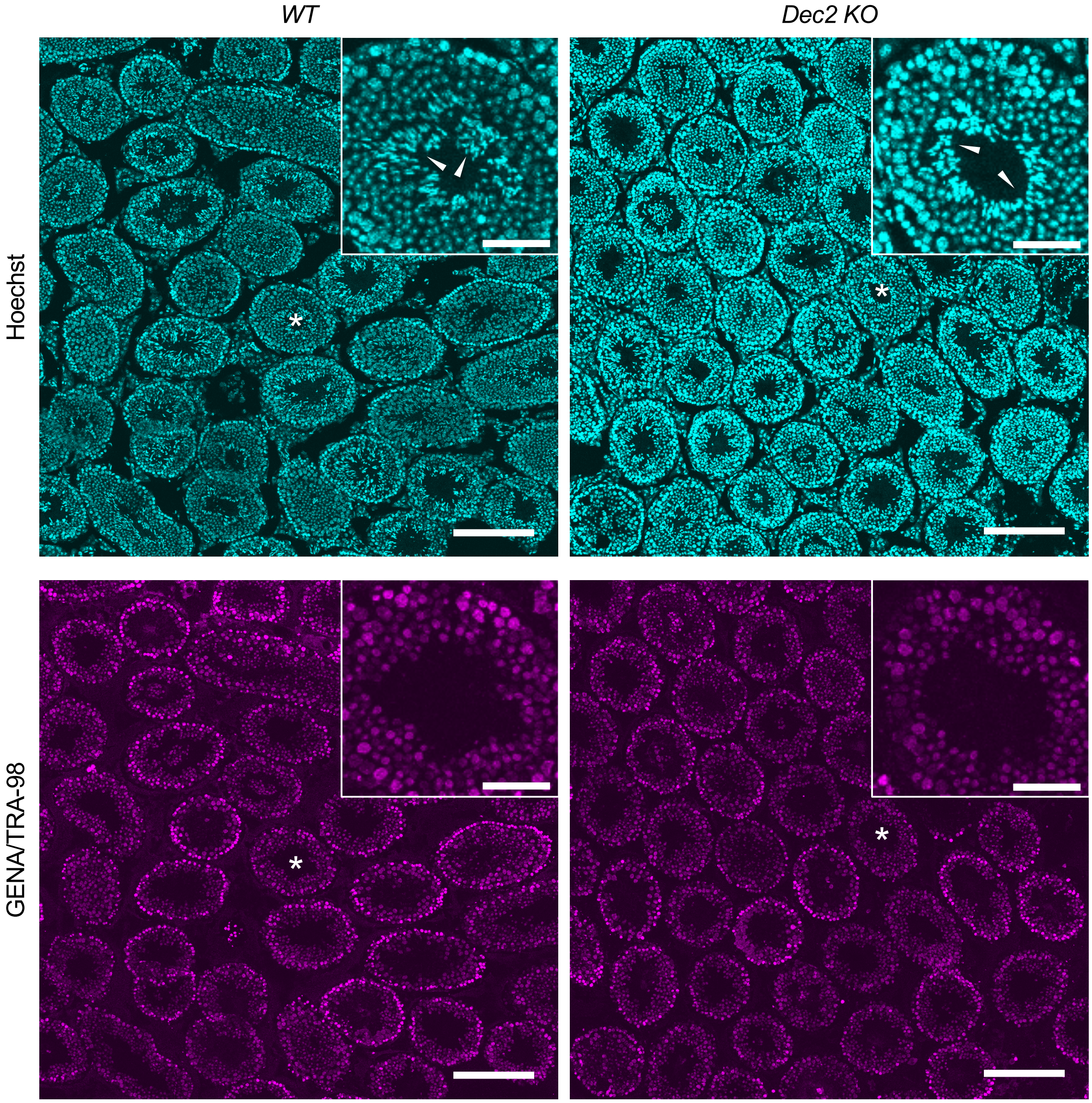

Supplementary figure S10 (related to Figs. 4a, 4b). Histopathological analysis of *Dec2* knockout testes.

Representative histological sections were stained using Hoechst (top panels) and anti-GENA/TRA-98 antibody (bottom panels). Asterisks indicate the tubules enlarged in the inlet images. White arrowheads indicate condensed spermatids, which were not stained anti-GENA/TRA-98 antibody due to the low accessibility of the antibody to the highly condensed chromatin. GENA/TRA-98, a germ cell marker. Scales, 200 µm and 50 µm (inlet images).

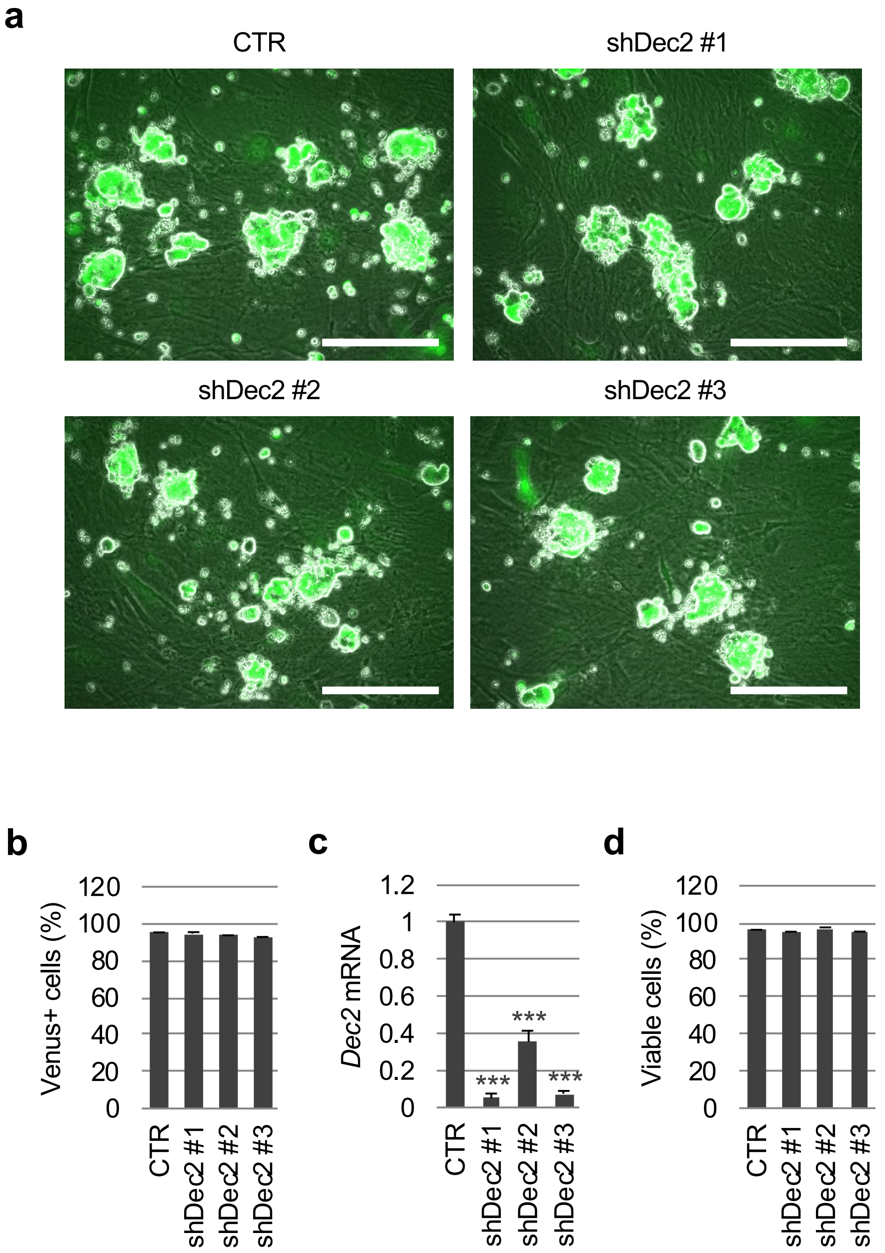

Supplementary figure S11 (related to Figs. 4d, 5a–c). Validation of *Dec2* knockdown by lentivirally transduced shRNAs in cultured SSCs.

(a) Representative images of the cultured SSCs at five days after lentiviral infection of shDec2 #1–3. Venus expression indicated the virus-infected cells. Venus fluorescent images were overlaid on the phase-contrast images. Scale, 200 µm. (b) Quantification of the lentiviral infection efficiencies. (c) Relative expression levels of *Dec2* were detected by RT-qPCR (CTR = 1). (d) Quantification of the viable cells after lentiviral infection. 7-AAD(-) cells were designated as viable cells. Dunnett's test was performed against CTR. Error bars indicate standard deviations. **p* value < 0.05; ***p* value < 0.01; ****p* value < 0.001. CTR, control; shDec2, shRNA against *Dec2*.

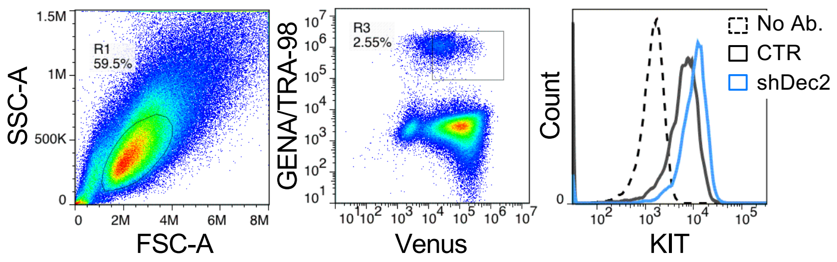

Supplementary figure S12 (related to Fig. 5d). Flow cytometric analysis to verify the increased expression of KIT by lentivirally transduced shDec2 to P3.5 neonatal germ cells. A representative result of flow cytometric analysis. R3 population, which was positive for both Venus and GENA, was recognized as lentivirus-infected germ cells, and their Kit expression levels were measured. No Ab., no antibodies.

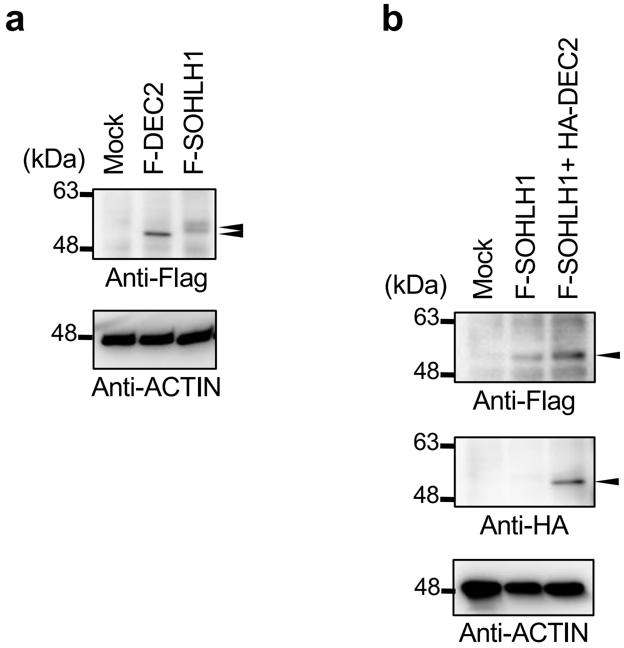

Supplementary figure S13 (related to Figs. 6b–d). Western blotting for the confirmation of exogenously expressed DEC2 and SOHLH1 in cultured SSCs.

(a, b) Arrowheads indicated the bands correspond to Flag- and HA-tagged DEC2 or SOHLH1. ACTIN was provided as loading control.

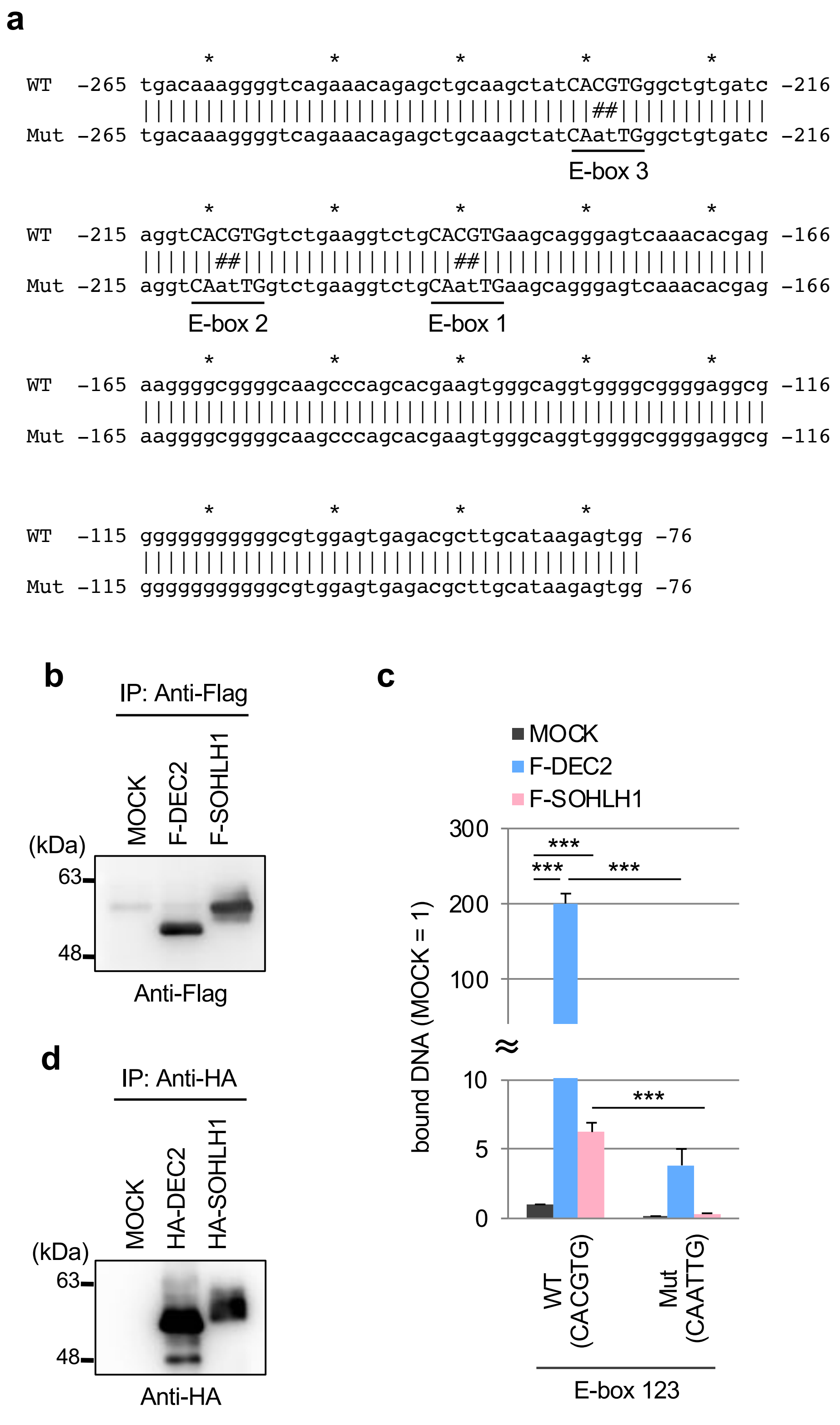

Supplementary figure S14 (related to Figs. 6a, 6e, 6f). *In vitro* promoter binding assay of DEC2 and SOHLH1 to E-box motifs in *Sohlh1* promoter.
(a) Sequence of *Sohlh1* promoter used for the DNA probe. Wild type (WT) and mutant (Mut) sequences of E-box 1, 2, and 3 are indicated with uppercase letters and lowercase letters, respectively. (b) Western blotting for the confirmation of the purified Flag-tagged protein beads. (c) *In vitro* promoter binding assay. The amounts of bound DNA were quantified by qPCR, and the results were indicated as relative values (Mock = 1). Dunnett's test was performed against F-DEC2 or F-SOHLH1 (three biological replicates). Error bars indicate standard deviations. ***p value < 0.001. (d) Western blotting for confirmation of the purified HA-tagged proteins.

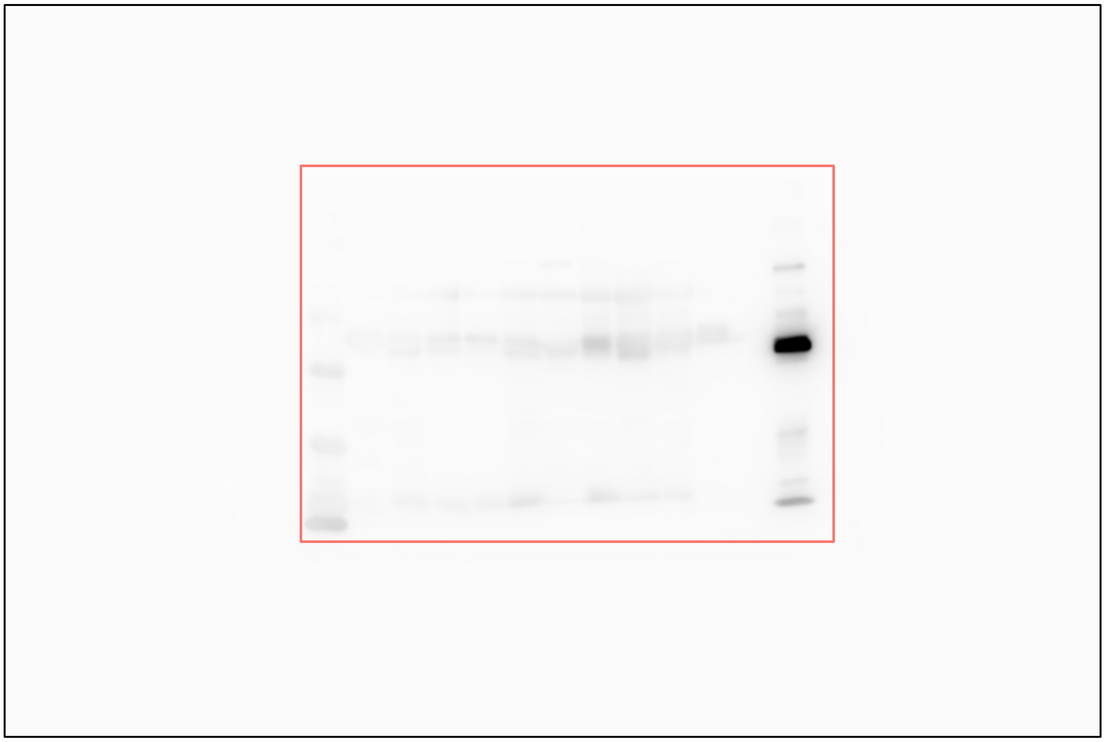

Anti-DEC2

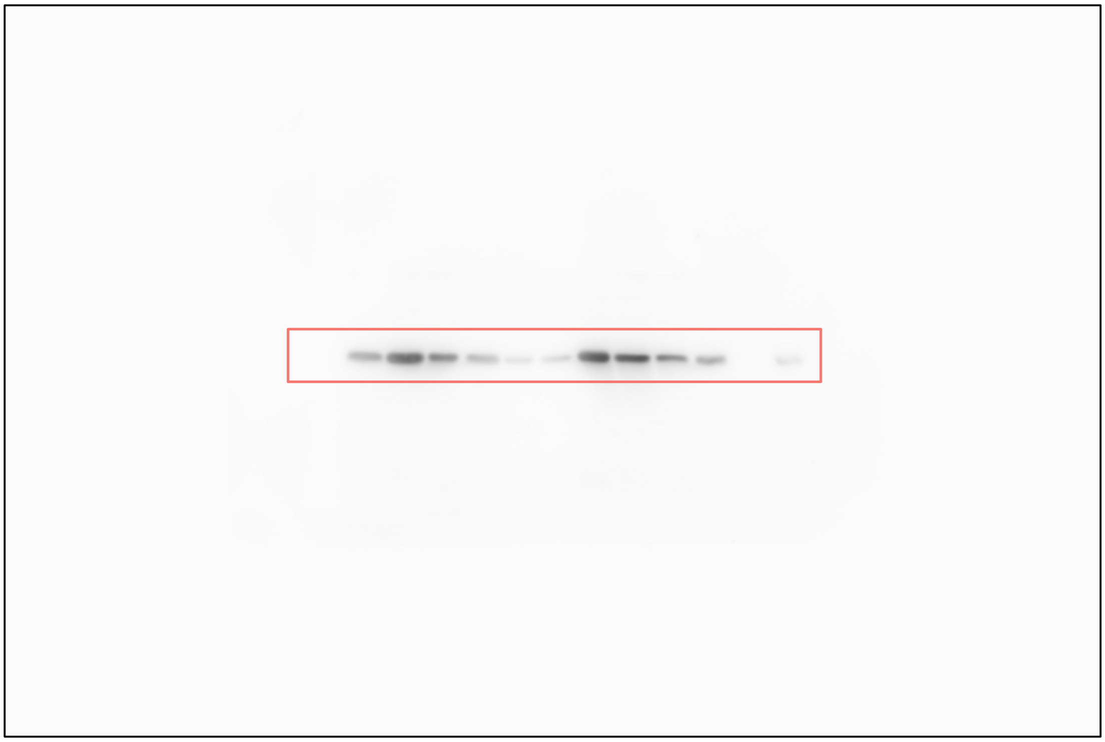

Anti-ACTIN

Raw images of Fig. S7a
Red squares indicate the cropped regions for the figure

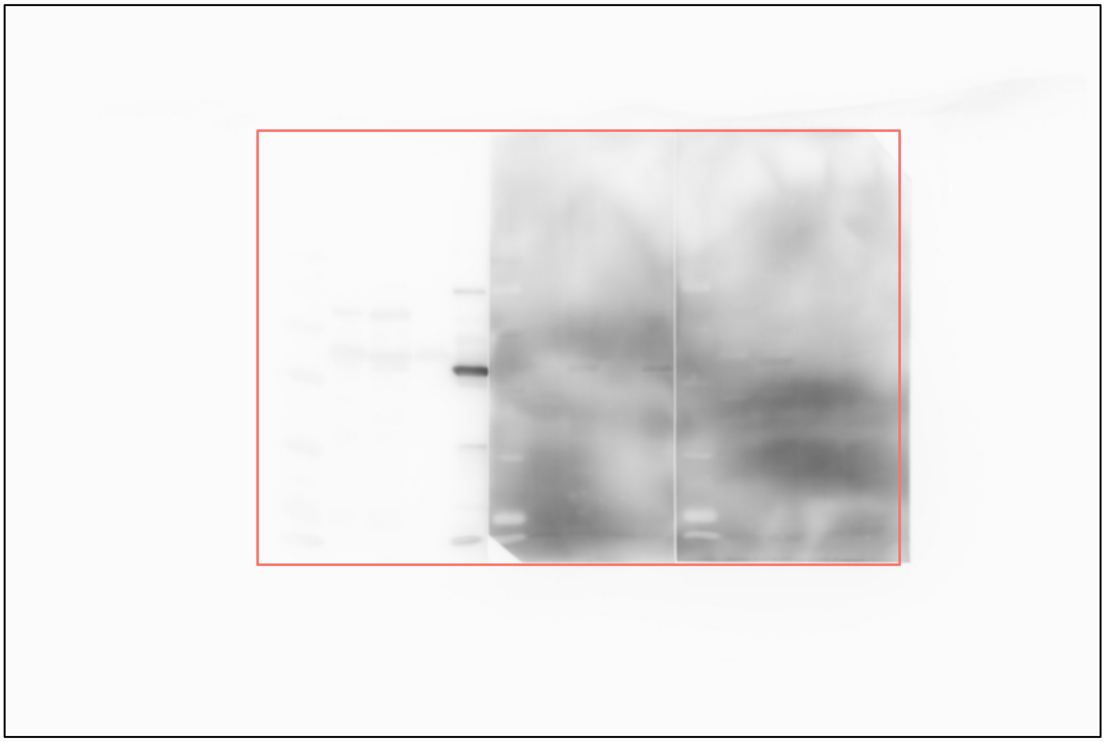

Anti-DEC2

Raw images of Fig. S7b

Red square indicates the cropped region for the figure

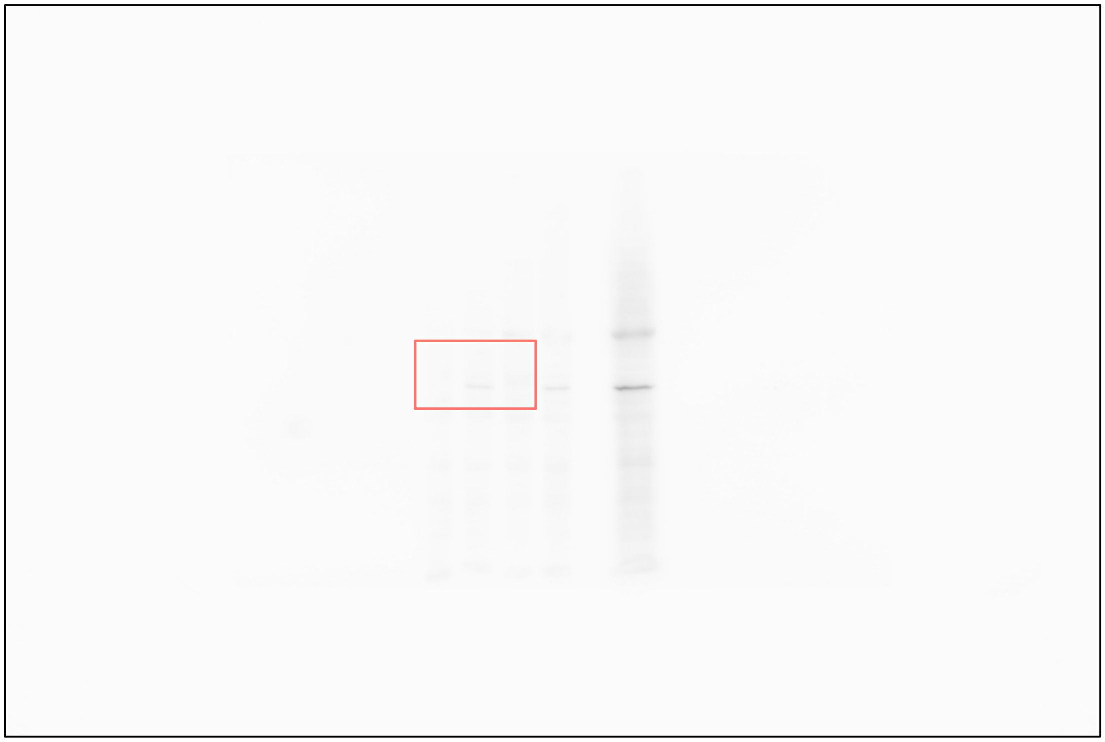

Anti-Flag

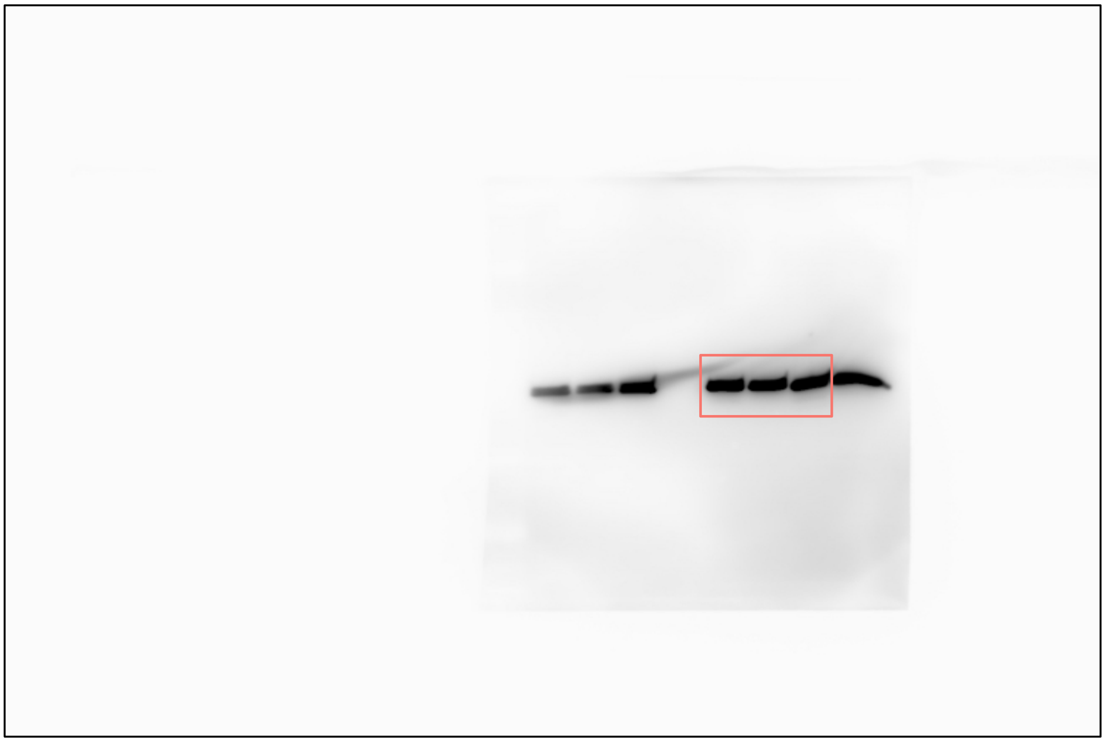

Anti-ACTIN

Raw images of Fig. S13a

Red squares indicate the cropped regions for the figure

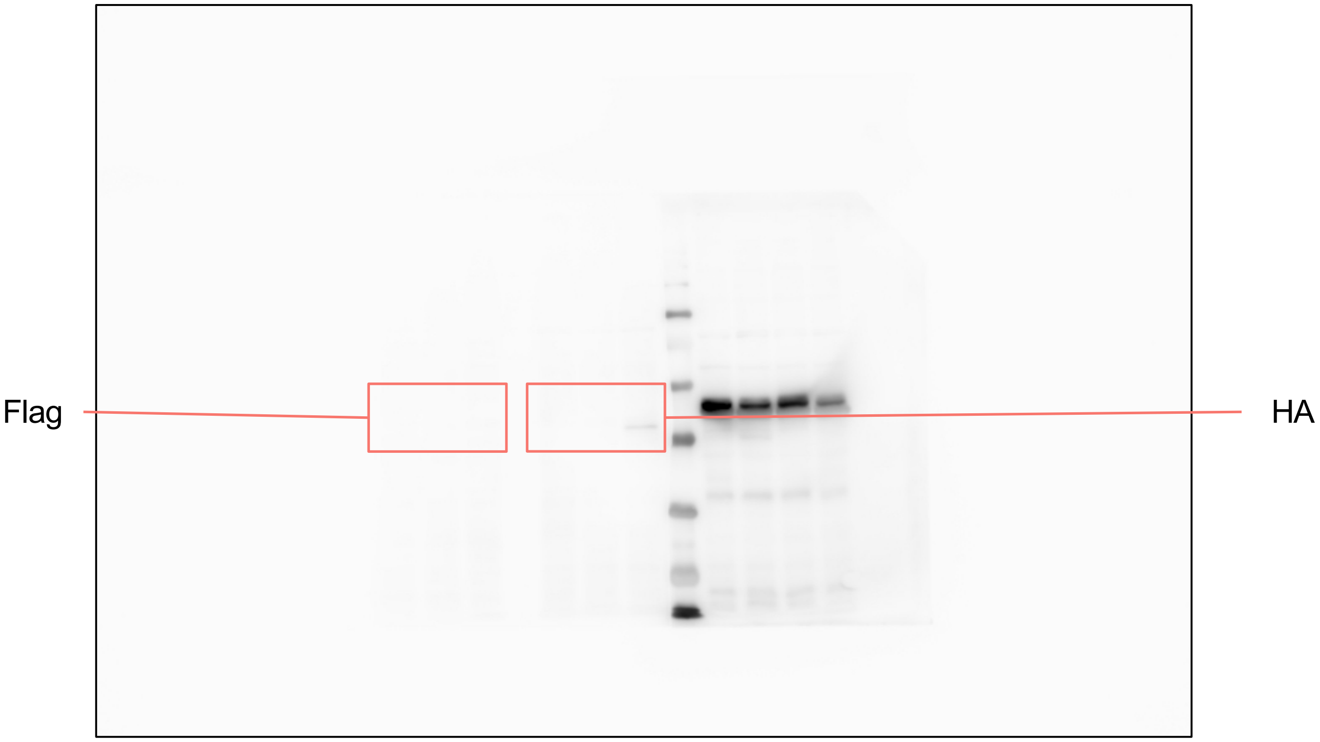

Anti-Flag/HA

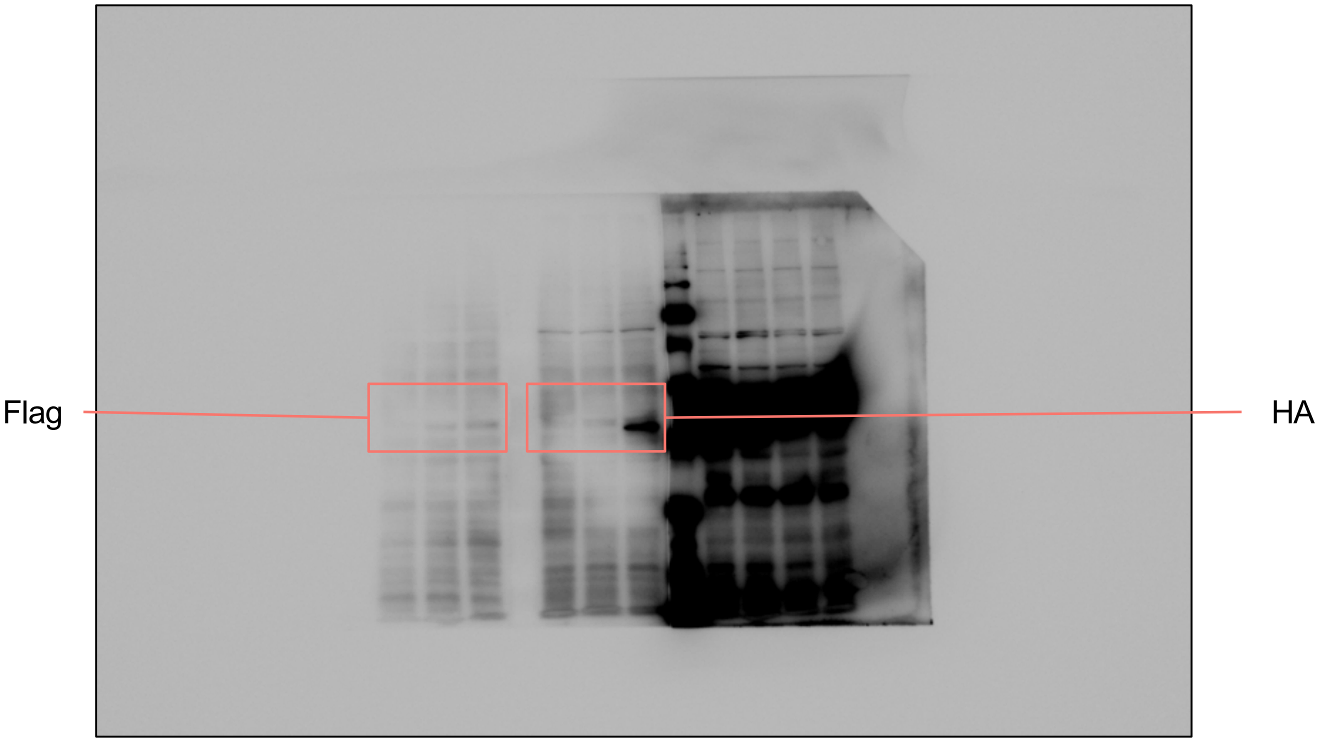

Anti-Flag/HA (Contrast-adjusted)

Raw images of Fig. S13b-1
Red squares indicate the cropped regions for the figure

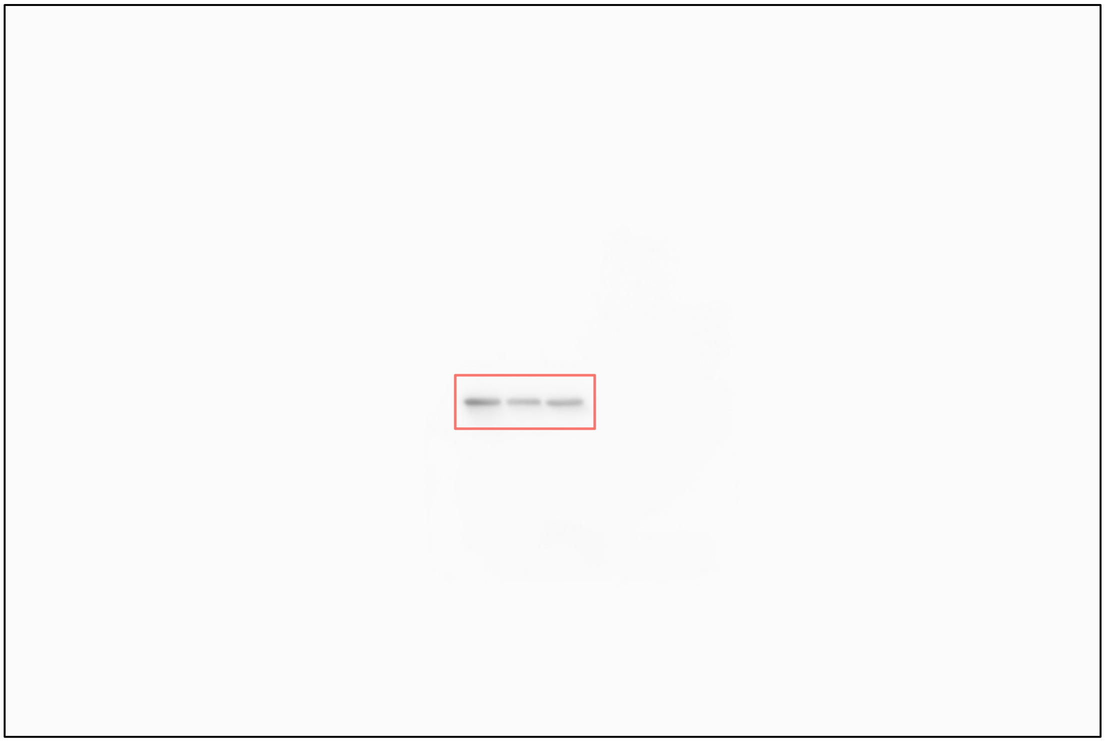

Anti-ACTIN

Raw image of Fig. S13b-2

Red square indicates the cropped region for the figure

b

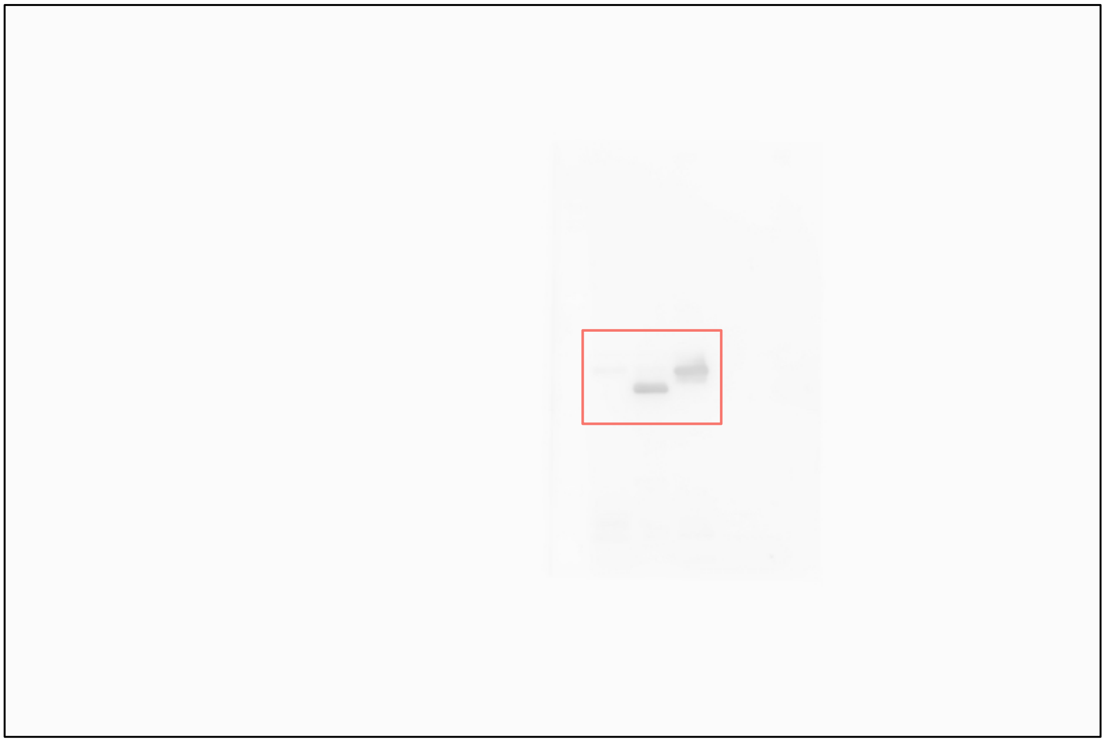

Anti-Flag

d

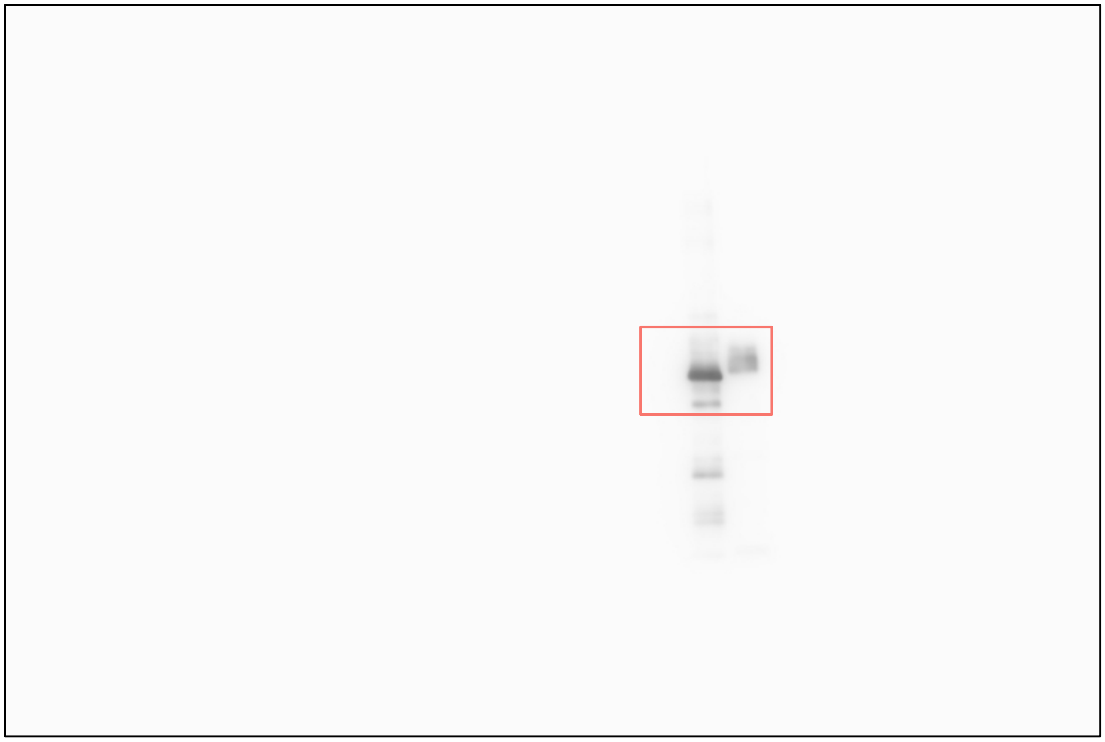

Anti-HA

Raw images of Fig. S14

Red squares indicate the cropped regions for the figure
